# CanRisk-GP protocol: A feasibility study of incorporating proactive multifactorial breast cancer risk assessment into general practice

**DOI:** 10.1371/journal.pone.0336902

**Published:** 2025-11-26

**Authors:** Francisca Stutzin Donoso, Stephanie Archer, Fiona M. Walter, Stephen Morris, Jon Emery, Jack Broome, Tim Carver, Joe Dennis, Lorenzo Ficorella, Amy Lafont, Adam E. Stokes, Laura Stylianou, Cameron Wilson, Douglas F. Easton, Marc Tischkowitz, Antonis C. Antoniou, Juliet A. Usher-Smith

**Affiliations:** 1 Department of Public Health and Primary Care, Primary Care Unit, University of Cambridge, Cambridge, United Kingdom; 2 Department of Psychology, University of Cambridge, Cambridge, United Kingdom; 3 Wolfson Institute of Population Health, Centre for Cancer Screening, Prevention & Early Diagnosis, Queen Mary University of London, London, United Kingdom; 4 Department of General Practice and Primary Care, Centre for Cancer Research, University of Melbourne, Australia; 5 Department of Public Health and Primary Care, Centre for Cancer Genetic Epidemiology, University of Cambridge, Cambridge, United Kingdom; 6 Department of Surgery and Cancer, Imperial Clinical Trials Unit, Imperial College London, London, United Kingdom; 7 Department of Oncology, Centre for Cancer Genetic Epidemiology, University of Cambridge, Cambridge, United Kingdom; 8 Department of Medical Genetics, National Institute for Health Research Cambridge Biomedical Research Centre, University of Cambridge, Cambridge, United Kingdom; All India Institute of Medical Sciences, INDIA

## Abstract

**Background:**

CanRisk is a risk assessment tool that implements the BOADICEA multifactorial breast cancer risk model. The BOADICEA model is recommended for use by the National Institute for Health and Care Excellence (NICE) in English, Welsh, and Northern Irish secondary/tertiary care to identify women who may be at moderate or high risk of developing breast cancer. BOADICEA combines information on cancer family history, demographic, lifestyle, hormonal risk factors and mammographic density with polygenic scores (PGS). Offering risk assessment using CanRisk in general practice has the potential to identify more women at moderate or high risk of developing breast cancer and improve their management and the appropriateness of referrals to secondary/tertiary care.

**Materials and methods:**

In this feasibility study we plan to invite women aged 40–49 years from 5–8 practices across Cambridgeshire and Peterborough in England, UK to complete a breast cancer risk assessment using CanRisk via a newly developed public-facing version of the CanRisk tool and provide saliva samples for PGS. The study team will provide a risk report back to both the participants and their GP, with those women at above-population level risk advised to make an appointment with their GP to be referred to the clinical genetics service and subsequently managed in line with current NICE guidelines. This study will provide evidence on (1) whether offering cancer risk assessment including PGS in general practice is feasible and acceptable to women and healthcare professionals; (2) whether this approach can identify women at above-population level risk of breast cancer who would otherwise not have been identified and so not had access to risk-reducing options; and (3) the costs associated with implementing proactive multifactorial breast cancer risk assessment in women under 50 within general practice.

**Study registration number:**

This study is listed on the ISRCTN registry. The registration number is ISRCTN17376192.

## Introduction

CanRisk is a cancer risk prediction tool that implements the Breast and Ovarian Analysis of Disease Incidence and Carrier Estimation Algorithm (BOADICEA) multifactorial model. This validated model combines information on family history, demographic, lifestyle, and hormonal risk factors, rare pathogenic genetic variants in cancer susceptibility genes, common genetic susceptibility variants summarised as a polygenic score (PGS), and mammographic density to estimate an individual’s future risk of developing breast cancer and of carrying a pathogenic variant in breast/ovarian cancer susceptibility genes [1–4]. CanRisk is regulated as an invitro diagnostic medical device in the UK and carries CE and UKCA markings. The National Institute for Health and Care Excellence (NICE) recommends BOADICEA and endorses CanRisk as a tool for assessing breast and ovarian cancer risk [5,6]. Depending on the degree of risk, women at above-population level risk are then potentially eligible for additional breast screening or risk-reducing medication [7].

Much research is being done to identify women over 50 years old who are at above-population level risk through the national breast cancer screening programme [8,9]. However, current guidance is that the selection of women under 50 years who should be referred for a risk assessment in secondary/tertiary care should be based on family history alone. This misses up to half of women who are at above-population level risk based on multifactorial risk assessment [10].

In June 2022, NICE also updated the guidelines on familial breast and ovarian cancer removing the recommendation against proactive assessment of breast cancer risk in general practice. This change was supported by a stakeholder consultation and opens up the potential for a general practice-based screening programme to identify those at above-population level risk of breast cancer rather than requiring women to self-present with concerns. This approach misses most women estimated to be at higher risk and increases inequalities [11]. A review of the literature against the consolidated framework for screening found that proactive risk assessment in general practice currently satisfies many of the standard principles for screening for women under 50 years [12]. However, while studies have demonstrated general acceptability (according to Sekhon et al [13]) and enthusiasm for assessing risk within general practice, previous research with the CanRisk tool has highlighted the need to tailor its development and use to the specific needs of general practice [14]. Research has also identified potential challenges to implementation, including the time needed to complete the assessment, IT requirements, competing demands and priorities, and training of staff [15]. It is not known whether multifactorial risk assessment will be acceptable to women within general practice or what the expected uptake would be and, despite rapid advances in understanding of the PGS contribution to risk assessment, few studies have attempted to implement PGS analysis within a general practice setting.

## Aims and objectives

The overall aims of this study are to evaluate the feasibility, acceptability and psychological impact of proactively offering multifactorial breast cancer risk assessment incorporating PGS using CanRisk in general practice, and to collect estimates of outcomes and data on costs and healthcare utilisation. The latter will inform the design of a future trial and a health economic analysis to model the estimated reductions in breast cancer incidence and mortality if proactive breast cancer risk assessment using CanRisk were to be introduced in general practice across the UK.

Specific objectives:

1. To quantify uptake of proactive multifactorial breast cancer risk assessment using CanRisk in general practice amongst women under 50;
2. To assess the acceptability of all stages of proactive multifactorial breast cancer risk assessment using CanRisk within general practice from the perspective of women, GPs, and staff in General Practice.
3. To compare the number of women identified as at moderate or high risk of breast cancer following proactive multifactorial breast cancer risk assessment with those identified through routine care and the number expected from population-based studies;
4. To quantify the psychological and behavioural impact of proactive multifactorial breast cancer risk assessment using CanRisk in general practice amongst women;
5. To quantify the amount of data collected via the MyCanRisk public-facing data collection tool within a general practice population;
6. To estimate the distribution of breast cancer risk amongst women under age 50 taking up the opportunity for multifactorial risk assessment following proactive invitation and uptake of risk-reducing interventions amongst those at moderate or high risk to inform sample size calculations for the design of a future randomised controlled trial;
7. To estimate differences in uptake of proactive multifactorial breast cancer risk assessment and breast cancer risk distribution of women between general practices to inform the design and sample size calculations for a future cluster-randomised controlled trial;
8. To collect data to enable estimation of the cost of delivering proactive multifactorial breast cancer risk assessment using CanRisk within general practice.

## Materials and methods

### Ethical considerations

REC approval from the Ethical and Health Research Authority has been granted by East of England – Cambridge Central Research Ethics Committee (REC reference 23/EE/0199).

Cambridge University Hospitals NHS Foundation Trust and the University of Cambridge are joint sponsors for this study.

Cambridge University Hospitals NHS Foundation Trust, as a member of the NHS Clinical Negligence Scheme for Trusts, will accept full financial liability for harm caused to participants in the study through the negligence of its employees and honorary contract holders. There are no specific arrangements for compensation should a participant be harmed through participation in the study, where no-one has acted negligently. The University of Cambridge will arrange insurance for negligent harm caused as a result of protocol design and for non-negligent harm arising through participation in the study.

### Design and setting

**Fig 1:**
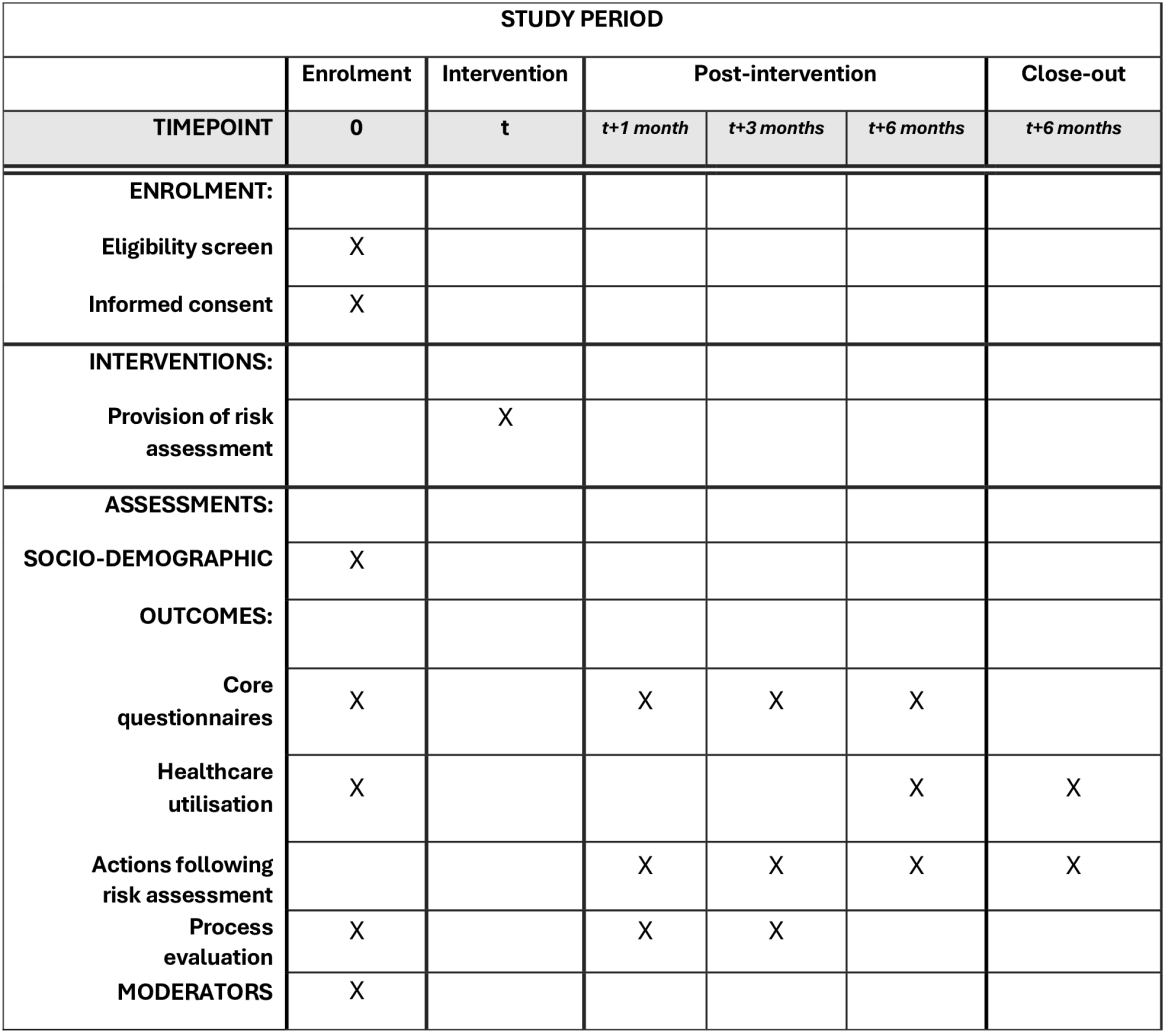
Study schedule of enrollment, interventions, and assessments according to SPIRIT 2013 requirements.

The study is a pragmatic multicentre feasibility study taking place in 5–8 GP practices in Cambridgeshire and Peterborough, England, UK. Fig 1 shows the study schedule (SPIRIT).

### Participants

The target population is women aged 40–49 years. The upper limit age of 49 years has been chosen as all women in England are invited to participate in the national breast cancer screening programme at age 50, and several studies are already in progress to evaluate the potential for offering risk assessment alongside mammography to identify women at above population risk [8,9,16].

An initial lower limit of age 40 years has been chosen because the main intervention amongst women at moderate and high risk of breast cancer is enhanced breast screening, and 40 is the minimum age in Cambridgeshire and Peterborough where women at moderate risk are eligible for this. If recruitment into the study is lower than expected, reducing the minimum recruitment age to 35 will be considered.

To be eligible for the study, women must also be registered with a participating general practice; and have capacity to consent and understand English.

Women are not eligible for the study if they have a breast cancer susceptibility gene already identified in their family or an entry in their medical records indicating any of: a personal history of breast or ovarian cancer, a known germline pathogenic variant in a breast cancer susceptibility gene, a previous diagnosis of metastatic cancer or entry on the palliative care register, or prior notification of dissent to research.

### Processes and interventions

#### Practice recruitment.

GP practice recruitment was supported by the Clinical Research Network (CRN) Eastern. They approached practices across Cambridgeshire and Peterborough that refer patients at increased risk of developing breast cancer to the clinical genetics service at the Cambridge University Hospital NHS Foundation Trust. Practices were recruited from different areas to maximise participant sociodemographic and ethnic diversity. Practices that agree to take part signed a practice agreement form. Reasons for practices declining to take part in the study were collected where possible.

#### Participant recruitment.

Eligible women are identified through searches of the electronic health records at General Practices based on the inclusion and exclusion criteria above. To prevent including women who may reach the age of 50 years between the searches being completed and receiving the invitations (see ‘Processes and Interventions’ section below), the search includes women aged 40–48 years. Depending on the population size of each practice, recruitment rate, and overall uptake into the study, either all or a random sample of those women who meet the eligibility criteria are invited to take part in the study. Invitations are staggered in random order. Those women are sent an invitation letter and S3 Participant information sheet by post. A reminder letter is sent by post two to four weeks later to those women who have not completed the consent form.

Women can complete the online written consent form which includes a screening question about breast cancer susceptibility gene already identified within their family. Those answering yes to this screening question are advised to make an appointment to discuss their breast cancer risk with their GP and are not able to continue with the study. After providing online written consent, all participants are asked to complete a short baseline questionnaire (details below) hosted online. On completion of the baseline questionnaire, participants are asked to provide data for a breast cancer risk assessment.

Recruitment opened on the 28^th^ of November 2024 for a duration of 8 months (until the 30^th^ of June 2025) or until 1,000 women have been recruited, whichever happens first.

**Fig 2:**
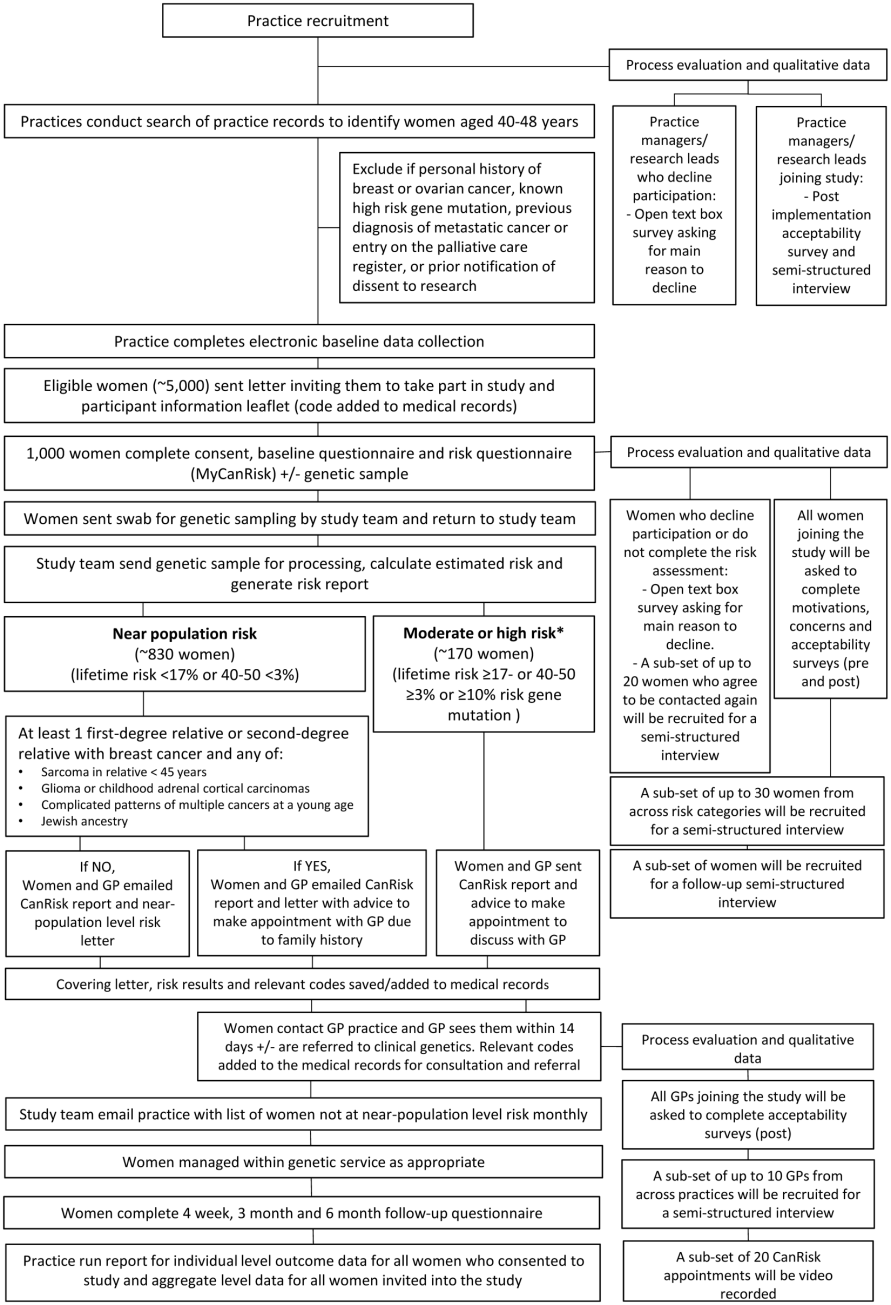
Study processes.

Participant recruitment and flow through the study is summarised in Fig 2.

#### Breast cancer risk assessment.

Personalised risk estimates are generated using the CanRisk tool. Population-based studies have shown that BOADICEA, including incorporation of polygenic scores, has moderate-to-good discrimination with AUCs or c-indices of 0.68–0.69 in women aged less than 50 years or those who are premenopausal [3,17] and is well calibrated with a ratio of expected to observed number of cases (E/O) of 0.97 [17].

For this study, personalised risk estimates are calculated based on family history, demographic, lifestyle and hormonal factors in addition to a PGS based on 309 common genetic variants [18]. Data on family history, lifestyle and hormonal factors is collected via the MyCanRisk app. Women receive a link to the MyCanRisk app after completing the consent and baseline questionnaire. MyCanRisk is a platform independent Progressive Web App that supports the on- and off-line collection of personal risk factor information alongside the detailed family history required to conduct a CanRisk calculation.

Participants have the option not to have PGS testing. For those participants consenting to this element of the study receive a saliva sampling kit on the post. The returned kits are collated by the research team and sent in batches for analysis by a clinically accredited laboratory. DNA samples which pass quality control are genotyped using a genome-wide array, specifically the Illumina Global Screening v4 Array (GSAv4). These data are then used (in conjunction with imputation to a reference panel to estimate genotypes for SNPs not on the array) to compute the 309 SNP PGS. The data is then sent to the bioinformatics provider for PGS analysis. The data is also used to estimate genetic ancestry: this enables the PGS to be standardised to the appropriate population and the use of ethnicity-specific BOADICEA model within CanRisk [19]. These results will then be returned to the research team.

The research team incorporates the PGS results together with the data on family history, lifestyle and hormonal factors collected via MyCanRisk, into the CanRisk tool and generate a personalised risk estimate for each participant. For those women opting not to have PGS testing, the personalised risk estimate is based on family history, lifestyle and hormonal factors only.

#### Risk categorisation and returning risk assessment results.

Based on the CanRisk breast cancer risk estimate and current NICE guidelines [7], participants are categorised into one of three risk groups: near population level risk, near population level risk but with additional features in the family history (i.e., sarcoma in relative < 45 years; glioma or childhood adrenal cortical carcinomas; complicated patterns of multiple cancers at a young age; Jewish ancestry), and moderate or high risk.

Women with an estimated breast cancer risk in the near population level risk group and without any additional risk factors based on their family history (see Table 1) receive a letter from the study team informing them that they are at near population level risk for breast cancer along with a breast awareness leaflet and the risk output from the CanRisk tool. The breast awareness leaflet is in line with current NICE guidance covering all the required fields (i.e., 5-point NHS plan for being breast aware, when to examine your breasts, what to look for, changes to look for, lifestyle advice, and further information). A copy of both the letter and risk output is also sent to the participants’ GP. Women with an estimated risk at near population level but with additional risk factors within their family history and those at moderate or high risk receive a results letter informing them that they may be at above average population level risk for breast cancer and advise them to make an appointment to discuss with their GP. They are also sent the breast awareness leaflet, and a copy of both the letter and risk output is sent to the participants’ GP. The GP or a delegate practice staff member adds the appropriate codes to the medical records for all participants.

**Table 1:** Risk categorisation.

Risk	Near population risk	Moderate risk	High risk
Lifetime risk between ages 20 and 80	<17%	≥17% - < 30%	≥30%
Risk between ages 40 and 50	<3%	≥3% - < 8%	≥8%

Women confirmed to have additional risk factors based on their family history or those at estimated moderate or high risk for breast cancer are offered referral to the regional clinical genetics service using a specially designed referral proforma. Women referred to the regional clinical genetics service are then managed in line with current practice.

Table 1 shows the risk cut offs for near population risk, moderate risk and high risk for lifetime risk (defined as the risk between the ages of 20 and 80), and 10 year risk between the ages of 40 and 50.[7] For women falling in different categories based on the two definitions of risk, whichever is highest determines the risk category (i.e., near population risk, moderate and high risk) they will be classified as for the purposes of implementing current NICE guideline [7].

A list of women advised to make an appointment with their GP is sent to practices each month. Any women who have not booked a consultation or already consulted with a GP is contacted by practice staff and supported to book an appointment if they wish. Each patient contact and any reasons for not wishing to consult with a GP is recorded by the practice staff.

The process for returning results to participants and subsequent management is shown in Fig 2.

#### Training to support GPs.

The research team provides training to GPs within each participating practice to increase GPs’ knowledge and confidence around multifactorial breast cancer risk prediction and help them conduct consultations with women within the study. The training is composed of six educational videos developed by the CanRisk research team and further optional educational resources (training videos available on www.canrisk.org). The content covered in the educational videos was developed following a scoping review of the training needs of healthcare professionals in general practice and feedback from members of the CanRisk general practice expert advisory panel [20].

## Study outcomes and measures

The study aims to generate data on the following outcomes:

- Uptake of multifactorial breast cancer risk assessment, including the proportion of those invited who consent and the proportion of those invited who complete the breast cancer risk assessment, with or without the inclusion of the polygenic risk score.
- Distribution of breast cancer risk, including the proportion of women who consent who are at moderate or high risk on the initial CanRisk assessment in general practice (with or without the PGS) and those at moderate or high-risk following assessment in secondary/tertiary care.
- Proportion of women at moderate or high risk of breast cancer. The proportion of registered women aged 40–48 years at the start of the study at participating practices who are at moderate or high risk of breast cancer after assessment in secondary/tertiary care will be compared with the proportion of women aged 40–48 years at moderate or high risk of breast cancer after referral to secondary/tertiary care at non-participating practices across Cambridgeshire and Peterborough during the same time period, and also compared with the proportion of registered women aged 40–48 years at participating practices in the 12 months prior to the study.
- Uptake of risk-reducing interventions, including the proportion of those at moderate or high risk who attend for a GP appointment, are referred to secondary/tertiary care, and take up enhanced screening and/or preventative medication.
- Completeness of risk factor information collected through MyCanRisk, including the presence of information completed for non-family history risk factors (e.g., height, weight, alcohol, women’s health) and the number of family members included in the pedigree.
- Change in psychological and behaviour measures (Table 2) following multifactorial breast cancer risk assessment at one month, three months, and six months after receiving the risk estimate.
- Acceptability of the pathway of proactive multifactorial risk assessment within general practice to women and GPs, including response rates and completion of questionnaires and the willingness for practices to take part.
- Costs and workload associated with delivery of proactive multifactorial risk assessment.

**Table 2:** Assessments.

Construct	Baseline	1 month	3 months	6 months
**Socio-demographic**				
Education status	•			
Postcode	•			
Family history				
Sarcoma in relative <45 years	•			
Glioma or childhood adrenal cortical carcinoma	•			
Multiple cancers in family at a young age	•			
Jewish ancestry	•			
**Outcomes**				
Risk perception				
Breast cancer risk perception (absolute)	•	•	•	•
Breast cancer risk perception (comparative)	•	•	•	•
Breast cancer risk conviction	•	•	•	•
Recall of risk information		•	•	•
Psychological				
Anxiety [22]	•	•	•	•
Cancer-specific worry [21]	•	•	•	•
Quality of life [24]	•	•	•	•
Impact of cancer risk assessment [27]		•	•	•
Satisfaction with test		•		
Views on the risk information [33]		•		
Attitude towards risk management options		•	•	•
Lifestyle and behavioural				
Current behaviour (breast self-examination)	•	•	•	•
Intention to attend screening	•	•	•	•
Change in behaviour (weight, alcohol consumption, exercise)		•	•	•
Healthcare utilisation	•			•
Actions following risk assessment (moderate/high risk or those with additional risk factors)				
Consultation with GP (or intention if not done)		•	•	•
Uptake of risk reduction strategies (or intention if not done)				
Enhanced screening		•	•	•
Risk reducing medication		•	•	•
Surgery		•	•	•
Process evaluation				
Acceptability [26]	•		•	
Motivations and concerns	•			
Views on GP consultation (only for moderate/high risk or those with additional risk factors)		•		
**Moderators**				
Numeracy [28]	•			
Health literacy [29]	•			
Beliefs about breast cancer [30]	•			
Time orientation [31]	•			
Intolerance of uncertainty [32]	•			

Table 2 specifies all measures, instruments and data collection points (baseline and at 1, 3 and 6 months after receiving the risk assessment results). All outcomes will be measured by self-report and include risk perception, psychological impacts of the risk assessment, lifestyle and behaviour, and actions taken following the risk assessment. Risk perception will be assessed using questions about absolute and comparative risk, risk conviction and recall of risk. Psychological measures include cancer-related worry, anxiety and quality of life using the Lerman cancer worry scale [21], the short-form of the state scale of the Spielberger State Trait Anxiety Inventory (STAI) [22] and the EQ-5D-5L [23, 24] respectively. These have been chosen as the Lerman cancer worry scale and short-form STAI have been widely used in the literature and so will allow comparison with other studies, and the EQ-5D-5L is the recommended measure for use in economic analyses by NICE [25]. We will also collect data on: motivations and concerns regarding participation in the study using multiple choice questions and short open text boxes questions; the acceptability of the study using 5-point Likert scale questions [26]; the impact of cancer risk assessment using the MICRA scale [27]; satisfaction with the test using 5-point Likert scale; questions assessing the degree of satisfaction with the multifactorial cancer risk assessment; views on the risk information using 5-point Likert scale questions assessing the information in the CanRisk report; views on the GP consultation, including assessing the usefulness of the consultation and whether the appointment clarified understanding of breast cancer risk. Additionally, patients who agree to have their appointment recorded will receive an optional survey immediately after their GP consultation exploring their experience of the appointment. Attitudes towards risk management options (i.e., earlier or more frequent screening, medication and surgery) will also be measured via standardised scales and multiple-choice questions reviewed by patient and public partners. Lifestyle and behavioural data will be collected through multiple choice questions; these will include question about current behaviour regarding breast self-examination, intention to attend screening, change in behaviour (i.e., weight, alcohol consumption and exercise) and healthcare utilisation (i.e., number and type of contact) specifically focusing on breast health in the six months prior to baseline and six months after baseline. Data on actions following the risk assessment will only be collected for women found to be at increased risk and if they have attended the GP appointment. Actions measured will include multiple choice questions about the attendance at the GP appointment (or intention if not done) as part of the study and uptake (or intention if not done) of risk reduction strategies (i.e., earlier or more frequent screening, medication and surgery). We will also collect data on numeracy [28], health literacy [29], beliefs about breast cancer [30], time orientation [31], and intolerance of uncertainty [32]. These will be considered as moderators for the measures above that will help test interactions between variables in the study.

### Data collection

The data collection process is shown in Fig 2.

Participant baseline data is collected via an online questionnaire.

Participant level follow-up data is collected via participant questionnaires at 1, 3 and 6 months after receiving the results letter and from the GP electronic medical records 6 months after the last participant has received the results letter for all individuals who have consented to participate in the study. This includes coded entries relating to breast symptoms, breast cancer risk and, for those at moderate and high risk, uptake of risk-reducing interventions.

Practice-level aggregated data on the age, ethnicity and Index of Multiple Deprivation of all eligible women will be extracted at the end of the study from the GP electronic medical records. Practice-level and aggregated individual level data on the number of referrals of women potentially at above population-level risk for breast cancer and the outcomes of those referrals for all practices across Cambridgeshire and Peterborough for the period of the study will also be extracted from the electronic health records at the clinical genetics clinic at Cambridge University Hospitals NHS Foundation Trust. For the practices participating in this study, the same data will be extracted for the 12 months prior to the study. Data on the number of women registered at each practice will be extracted from publicly available National General Practice profiles.

A mixed-methods process evaluation is being conducted alongside collection of outcome data to explore how proactive breast cancer risk assessment is delivered and perceived at practice and patient levels. The main four questions guiding this element of the study will focus on: 1) whether this feasibility study did what it was set out to do; 2) whether the different elements in the study design worked and why; 3) what the different stakeholders thought about CanRisk and how it was implemented in general practice: and 4) whether implementing CanRisk in general practice is acceptable. Data is collected from women, GPs conducting breast cancer risk consultations, and practice managers and/or research leads from the different research sites. Three sources of data contribute to this process evaluation.

#### Questionnaires.

The baseline questionnaire for women includes questions covering participants’ motivations and concerns regarding their participation in the study and its prospective acceptability [26]. The one-month follow-up questionnaire for those advised to have a consultation with their GP includes questions to assess participants’ views on the usefulness of the consultation, and whether the appointment clarified their understanding of breast cancer risk. The three month follow-up additionally includes questions about retrospective acceptability [26]. All GPs taking part in this study will be invited to complete an electronic questionnaire after the study. The questionnaire includes questions around acceptability [26] and from the NoMAD checklist about the potential for the intervention to be incorporated into practice [34]. One practice manager and/or research lead per research site will also be invited to complete the NoMAD survey completed by GPs.

#### Qualitative interviews.

A sub-set of up to 30 women who complete the risk assessment will be recruited to take part in a semi-structured interview lasting up to one hour and covering their views on the entire pathway. We will also ask for verbal consent from women attending an interview for us to potentially contact them about an optional follow-up interview up to six months after the initial interview to gain more in depth understanding of later aspects of the pathway. This sample size reflects the aim of this element of the study, namely to inform the process evaluation and takes into account feasibility and capacity aspects. We will aim to interview women at different levels of risk and, if possible, women from heterogeneous socio-economic backgrounds.

Women who decline to take part in the study are asked to provide a one sentence description of their main reason not to join (decliner form). Women who agree to take part in the study but decline PGS testing are also asked to provide a one sentence description of their main reason to decline PGS testing. If possible, a sub-set of up to 20 women who decline or do not complete specific aspects of the study, including those who do not complete the risk assessment or decline taking part in the study, will be recruited to take part in a semi-structured interview to shed light on potential barriers for future uptake.

A sub-set of up to 10 GPs from all practices will take part in a semi-structured interview lasting up to 30 minutes to learn about their views on the intervention; their participation in the study; their experience completing the CanRisk training; their thoughts about the CanRisk report used to discuss the risk assessment results with women; and time/costs associated with the implementation of CanRisk.

One practice manager and/or research lead per research site will take part in a semi-structured interview lasting up to 30 minutes to gain more in depth understanding about how the study was implemented in their practice and the time/costs associated with the implementation of CanRisk. Practice managers and research leads who decline the invitation for their practices to join the study were also asked to provide their main reason for declining. These data were collected by the Clinical Research Networks in an online Google form.

For women who decline participation in the study, a participant information sheet and consent form for the interviews is provided following the decliner form. Separate consent will also be taken prior to all other interviews. Interviews will be conducted either face to face or online depending on the feasibility and interviewees’ preferences.

#### Video recording of consultations.

A sub-set of 20 GP appointments will be audio- or video-recorded to reflect on how risk, management options and next steps are discussed in practice as well as time spent on CanRisk appointments. Information about this is included in the breast cancer risk assessment results letter for women at increased risk and those interested in this element of the study are asked to contact the study team once they have made an appointment at their GP surgery. Still, between three and five days after women receive the results letter, the study team will follow up with those who do not get in touch spontaneously to confirm their interest. E-consent from women and healthcare professionals to audio or video record appointments will be sought before the appointment. Continued consent will be checked at the beginning and end of the consultation by the GP.

### Sample size

Based on an uptake of 16% in the only study in the UK that invited women to complete a breast cancer risk assessment [35], we estimate that to recruit 1,000 women we need to invite approximately 5,000 women from across the GP practices.

Based on distributions of known risk factors in the UK, we estimate that approximately 17% (170) of those 1,000 women will be at above population-level risk [1]. This will allow us to estimate:

- the proportion of eligible women in each practice who consent to take part in the study with an accuracy of +/- 1.1% (width of the 95% CI) based on an estimate of 20%
- the proportion of eligible women in each practice who consent who complete the risk assessment with an accuracy of +/- 2.5% based on an estimate of 80%
- the proportion of women who consent who are at moderate or high risk on initial CanRisk assessment in general practice with an accuracy of +/- 2.5% based on an estimate of 17%
- the proportion of those at moderate or high risk who attend for an appointment with a GP and who are referred to secondary/tertiary care with an accuracy of +/- 5.7% based on an estimate of 80%

Allowing for a 40% loss to follow-up, 1,000 participants will allow us to detect a change in anxiety or worry of 0.5 SD or greater at each time point at p < 0.05 and 90% power in both women at population level risk and in the subgroup identified at moderate or high risk. 1,000 participants will also allow us to detect an estimated increase in the proportion of registered women identified at moderate and high risk through proactive multifactorial risk assessment across all the participating practices compared with the 12 months prior to the study with a 95% confidence interval of +/- 0.005 (5 per 1,000 registered women).

Together with data on the acceptability of the proactive multifactorial breast cancer risk assessment, the following progression criteria will be evaluated to judge whether a subsequent full trial of proactive multifactorial breast cancer risk assessment would be feasible: 16% uptake into the study; 60% response to follow-up questionnaires; 80% of women receiving their breast cancer risk and offered subsequent risk management advice/referral. If any of those criteria are not met, we will use the data from the process evaluation to adapt the approach for subsequent feasibility and/or pilot studies before consider progressing to a subsequent full trial.

### Analysis

#### Quantitative data analysis.

Descriptive statistics will be used to summarise characteristics of the eligible population and study population at baseline, recruitment and retention rates, satisfaction and understanding of test results and consultations, healthcare utilisation, and intention to change behaviour overall and by risk group. Means and standard deviations will be used for normally distributed continuous variables, medians and interquartile range for non-normally distributed continuous variables, and numbers and percentages for categorical variables. Where reported, all proportions and differences in proportions will be presented with 95% confidence intervals.

#### Uptake of breast cancer risk assessment.

Logistic regression will be used to assess the participant level characteristics associated with completion of the risk assessment amongst those consenting to the study, reporting both unadjusted and adjusted odds ratios (OR) with 95% confidence intervals. Chi-squared tests will be used to compare the proportions of those invited who take up the offer of breast cancer risk assessment between demographic subgroups using aggregate data from each participating practice. Test significant at p < 0.05 will be presented as statistically significant.

#### The quantity of self-reported data using MyCanRisk.

The amount of information completed for non-family history risk factors alongside the number of family members reported in the pedigree will be summarised using descriptive statistics. Chi-square tests will be used to assess differences in the amount of information provided between demographic subgroups.

#### Psychological and behavioural impact of multifactorial breast cancer risk assessment.

For continuous outcomes (anxiety, worry, risk perception, recall of risk information, and intentions) analysis of covariance will be used to calculate change at each follow-up within each risk group, adjusting for clustering between each GP practice. Any differences between the risk groups on analysis of covariance will be tested using an F-test, followed by estimation of the comparison between those at near population level risk and those at moderate or high risk based on the CanRisk assessment. All models will be adjusted for age, ethnicity, education level, deprivation, baseline EQ-5D and general practice. The size of the change between groups will be interpreted by comparison with the standard deviation (SD), with the criteria for clinically relevant change being a change of 0.5 SD [29]. We will also test for interactions between the risk groups and age, family history of breast cancer, numeracy, beliefs about breast cancer, time orientation, intolerance of uncertainty and responding “not anxious or depressed” versus “slightly/moderately/severely or extremely anxious or depressed” on EQ-5D at baseline. Analyses will be repeated within the subgroups where the p value for interaction is < 0.05.

#### Process evaluation.

Qualitative data from semi-structured interviews will be analysed using inductive and deductive thematic analysis [36]. All interviews will be audio recorded and transcribed verbatim before analysis. The inductive analysis will look for emergent themes relevant to the study aims and the deductive thematic analysis will be organised around the seven dimensions of the Theoretical Framework of Acceptability [13]. According to this framework, acceptability is defined as a “multi-faceted construct that reflects the extent to which people delivering or receiving a healthcare intervention consider it to be appropriate, based on anticipated or experienced cognitive and emotional responses to the intervention” [13]. Findings from interviews with participants, non-participants and clinicians will be compared, looking for concordant and discordant themes. A fidelity checklist based on the training provided to GPs will be used alongside the video recordings from healthcare appointments to assess the adherence of GPs to the protocol during consultations with women at moderate or high risk. Verbatim transcriptions from the audio of the video recordings will be analysed qualitatively using thematic analysis to explore challenges and strengths in GP communication, patient reactions, and patient-clinician dynamics. Coding will be performed deductively by two independent researchers.

Descriptive statistics will be used to analyse the results from the questionnaires. Content analysis will be used to analyse the short open text responses from non-participants (women and practice managers and or research leads).

#### Economic analysis.

Unit costs from published sources [37–39] will be applied to healthcare utilisation data and used to calculate costs per participant in the 6 months before and the 6 months after baseline. This includes the time and costs associated with using the cancer risk assessment. Costs and resource use will be reported as means, standard deviations, medians, and interquartile ranges. Data from the study will be applied to an existing cost-effectiveness model [40] to evaluate the cost-effectiveness of the intervention.

## Results

Results on the primary outcome of this study are expected by the 31^st^ of August 2026.

### Project management and co-ordination

#### Study management.

The core study management team will meet regularly throughout the study. Additional meetings between core study management team members and the wider research team will be arranged as required. The study coordinator and study manager will lead the communications with the research sites and support the overall management of the study including protocol amendments. Key co-investigators will be invited to monthly Trial Management Group meetings.

#### Safety considerations.

Related Serious Adverse Events (SAEs) will be reported from the point of participant consent until the time that their breast cancer risk result letter is sent to them. Causality will be assessed by the practice PI (or delegated, appropriately trained investigator) based on all available information at the time of the completion of the SAE report. There are no expected SAEs for this study.

Any SAEs that are deemed to be unrelated to the study participation will not be reported. Adverse events will not be reported by sites.

#### Data management.

All data management is compliant with current UK data protection regulation GDPR. Identifiable participant data received electronically will be held in strictest confidence by the research team at University of Cambridge, with access limited to the research team by two-factor authentication. Data will be stored on the University of Cambridge Secure Research Computing Platform (SRCP). SRCP is ISO 27001 certified and is NHS Security and Protection Toolkit compliant.

Data transferred will not contain personal identifiable information except for communications between the research team and the professional company conducting DNA extraction and analysis (who will have signed a confidentiality agreement) and GP surgeries, and communications within the research team.

Pseudo-anonymised data will be transferred electronically between members of the research team, following local data security policies. Transfer of personal data within the research team and between the research team and GP surgeries will be done via NHS.net email.

The recordings and anonymised transcriptions from interviews and consultations will initially be saved onto a University of Cambridge encrypted computer and then uploaded to the University of Cambridge SRCP. After being uploaded to the SRCP, the local copy will be deleted. For transcription. recordings will be securely uploaded (via SFTP or other encrypted method) to an external transcription company who will have signed a confidentiality agreement.

If participants wish to withdraw from the study no further data will be collected on them, though we will keep all data collected to that point. Participants will be free to withdraw consent during the interviews without giving a reason. They will be asked if they consent to the research team using the data collected prior to withdrawal. All participants withdrawals will be recorded.

Any personal data not needed for future research, will be destroyed 12 months after the end of this study.

### Patient and Public Involvement and Engagement

This study has been designed in collaboration with the CanRisk PPIE group. The CanRisk PPIE group includes fourteen patient/public partners. To date, our patient/public partners have been involved in securing funding, designing the study and reviewing the study documentation. Over the course of the study, we will meet with our PPIE group on a regular basis, along with our general practice advisory group, which is composed of three nurse practitioners and seven general practitioners working in general practice across England. We will also seek additional advice on issues that arise throughout the study as necessary. Once the data collection is complete, we anticipate that our PPIE group will support data interpretation following initial analysis and will help to disseminate the findings once the study is complete.

#### Study status and timeline.

The study is currently at the stage of participant recruitment. Recruitment of participants to the study will run from 28 November 2024–30 June 2025, or until 1,000 women have been recruited. Data collection will continue for 6 months after the last recruited participant has received their risk estimate.

## Discussion

This is the first study to evaluate the feasibility, acceptability and psychological impact of incorporating proactive multifactorial breast cancer risk assessment into general practice for women aged 40–49 and one of the first to assess the feasibility and acceptability of incorporating polygenic risk scores within risk assessments conducted within general practice. As such, it will not only provide important data on the potential of adopting a proactive approach to breast cancer risk assessment in this setting but will also generate data to inform the design of future studies on the implementation and clinical utility of using PGS more generally within general practice.

Furthermore, this work will contribute towards building the evidence for future larger scale research on the uptake of risk-reducing options among women at moderate and high risk of breast cancer and health economic analyses to model the estimated reductions in breast cancer incidence and mortality if multifactorial risk assessment using CanRisk were to be introduced in general practice across the UK. The results should also inform similar implementation in other countries.

Findings from the study will be reported in open-access papers in peer-reviewed journals and presented at national and international conferences. We will also provide a lay summary of the findings on the study website.

## Supporting information

S1 File Participant information sheet. (DOCX)

S2 File SPIRIT_Fillable-checklist-15-Aug-2013. (DOC)

S3 File CanRisk GP protocol Nov 2025. (PDF)
