# Supplementary material for: CanRisk-GP protocol: A feasibility study of incorporating proactive multifactorial breast cancer risk assessment into general practice
Source: PLoS One. 2025 Nov 26;20(11):e0336902. doi: 10.1371/journal.pone.0336902 (PMC12654911; doi:10.1371/journal.pone.0336902)
Supplement: S1 File — (DOCX) [file pone.0336902.s001.docx]

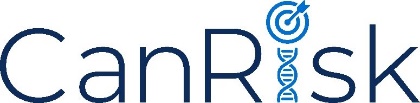


**CanRisk-GP: a feasibility study of in****corporating multifactorial breast cancer risk assessment into general practice**

**PARTICIPANT INFORMATION SHEET**

# You have been invited to take part in the CanRisk-GP study. Please read the following information carefully. This will help you to decide on whether you want to take part in the study or not. There is no obligation to take part, and if you wish not to, the care you would normally receive from your GP and from other NHS organisations will not be affected in any way.

# If anything is not clear, or you would like more information please get in touch using the contact details provided at the end of this leaflet.

After reading the information sheet, you can either sign up to the study or let the research team know why you can’t or don’t wish to take part by going to the study website using the link in your letter of invitation.

# ___________________________________________________________________

**Summary of the study:**

What is the purpose of the study?

Breast cancer is common in the general population. The estimated risk of being diagnosed with breast cancer over the life course is 1 in 7 (15%) for females in the UK. For women aged 30 or above who are at increased risk of developing breast cancer there are options available to reduce this risk and help find any cancers that do develop early. These options include making changes to their lifestyle, earlier screening and medication. Some women learn about their increased risk of developing breast cancer because of other cancers in their family. Women without a family history of breast cancer can also be at increased risk. We know that there are many women who do not know that they are at increased risk and they are therefore missing out on potential options to reduce it.

The CanRisk tool combines genetic, family history, lifestyle and hormonal risk factors about women to help doctors predict each woman’s future risk of developing breast cancer. CanRisk is widely used in specialist clinics with women who are known to have an increased risk, but it has not yet been implemented in general practice, where it could help identify more women who are at increased risk of developing breast cancer. Very little is known about whether and how to start using CanRisk in general practice.

In this study, we are offering women the opportunity to complete a CanRisk assessment and receive an estimate of their risk of developing breast cancer. Women found to be at increased risk of developing breast cancer will be advised to contact their GP to discuss what steps they can take to reduce their risk. If appropriate, and with their agreement, they will then be referred to specialist care at the Clinical Genetics Service in Addenbrooke’s Hospital Cambridge to see if they are eligible for earlier screening or medication. We will measure women’s interest and availability to learn about their breast cancer risk, ask them about their experience of the study and assess the consequences that undergoing this assessment may have for them. We will also reflect on how GP appointments go and ask GPs, as well as other practice staff, what they think of CanRisk and how it works in general practice. This will help us find the best way to reach more women at increased risk of developing breast cancer and offer them ways to reduce it.

**Who is taking part?**

We are inviting women between 40 and 49 years old who do not already know they are at increased risk of developing breast cancer, have not already been diagnosed with breast or ovarian cancer, have not opted out of being contacted for research and are registered at GP practices across Cambridgeshire and Peterborough to take part in this study. If we can’t find enough women within this age group, we may also invite women between 35 and 40 years old. We have chosen these age limits because women between 35 and 49 years old who are at increased risk of developing breast cancer are eligible for risk reducing options in Cambridgeshire and Peterborough. We are also asking the healthcare professionals and practice managers involved in the delivery of CanRisk assessments to take part in an interview at the end of the study.

**Do I have to take part?**

# No. It is entirely up to you to decide whether or not to take part.

# If you decide not to take part, we would be interested in your reasons for that. If you wish to, please go to the study website using the link in your letter of invitation to tell us your reasons in a few words. Your reasons not to take part are very valuable, as they can inform research and future care.

**What will happen if I decide to take part?**

- Consenting to take part

If you would like to take part in the CanRisk-GP study, we will ask you to complete an electronic consent form. It is important to know that we are only collecting essential personal and research data from you that is necessary for us to meet the aims and objectives of this study. If, for any reason, you are unable to complete the consent form electronically or need assistance, the research team will be happy to help you. Contact details are at the end of this document.

- Completing some questionnaires and MyCanRisk

After you have completed the consent form, the research team will send you a questionnaire to complete. Our preference is to send these electronically but you will have the option to select to receive them by post. The questionnaire will include multiple choice questions about you and your background, your thoughts about breast cancer and risk more generally, as well as your health and wellbeing. We anticipate this will take you around 20 minutes to complete. You will also receive a link to MyCanRisk, which will ask you information about yourself and your family to calculate your breast cancer risk. We anticipate this may take you around 30 minutes to complete, but this is only an estimate. It is hard to know exactly how long it will take you to complete MyCanRisk because it will mainly depend on the size of your family. As this is also completed electronically, you can save it and come back to it as many times as you need.

- Completing a saliva sample kit at home

You will be asked if you would like to include genetic information in your assessment. Most of the genetic code is the same between individuals. However, there are small genetic differences between each of us that are present from birth and do not change throughout your life. Some of these differences increase the risk of developing breast cancer and some of them decrease the risk of developing breast cancer. By looking across all the small differences, we can calculate something called “polygenic score” for breast cancer risk. Calculating your polygenic score is a way of comparing your risk with the risk of people who have a different genetic constitution. Combining your polygenic score with information about you and your family will enable us to make a more accurate estimate of your risk of developing breast cancer.

If you agree to include genetic information in your assessment, we will send you a saliva sample kit at your preferred address for you to complete. We will send you a pre-paid envelope for you to use to post it back to us. You will be asked to confirm your details on a participant information slip and include this in the box with your saliva sample. Once the information from MyCanRisk and the saliva sample have been processed you and your GP practice will receive a letter from the research team with an electronic report of your risk assessment results. The polygenic score informed in your report will not have any implications for your family.

As well as these small differences in the genetic code between individuals, there are also specific genes, such as the *BRCA1* gene, that run in families and have a big effect on people’s risk of developing breast cancer. We will not be testing for these genes in the saliva sample you provide.

- Receiving your results

You will receive your results by email within 16 weeks of consenting to take part.

- - If you are NOT at increased risk of developing breast cancer

Most women joining the study will be at population risk, which means they do not have an increased risk of developing breast cancer. If you are found to be at population risk, you will receive your CanRisk report and a results letter including breast awareness information. You will not need to see the GP.

- If you ARE at increased risk of developing breast cancer

If you are found to be at population risk but have told us about information in your medical history that might affect your risk, or your results show you are at moderate or high risk of developing breast cancer, you will receive your CanRisk report and a results letter and will be advised to book an appointment with your GP to discuss the results.

This appointment will take place no longer than 2 weeks after you contact your GP practice and will have a normal duration of up to 15 minutes. During the appointment, your GP will talk through your risk with you and what you can do about it. If appropriate, you will be offered a referral to a specialist for a follow-up.

- Completing some follow-up questionnaires:

Regardless of your risk results, you will receive links to complete follow-up questionnaires after 1, 3 and 6 months of completing the CanRisk assessment. The questionnaires will include multiple choice questions about your thoughts on the result of your risk assessment, breast cancer and risk more generally, as well as your health and wellbeing. We anticipate these will take you around 20 minutes each time.

**Additional (optional) research activities**

- Interviews

We would also like to talk to a small number of participants individually at a later date to discuss their thoughts about taking part in this study. You will be asked at the beginning of the study whether you would like to take part in this. The conversation can last for up to 1 hour and the details will be arranged with you to accommodate your preferences and availability as much as possible. The conversation can be online or in person at your home or at the University of Cambridge. If the interview takes place at the University of Cambridge, we will reimburse your travel costs. With your agreement we will audio record the interview. If the interview is completed online, you will have the option to turn your camera off for the recording.

- Video recording of consultations

For those that are asked to attend an appointment with their GP, we would like to video and audio record a small number of those appointments. You will be able to decide before the consultation if you are happy for your appointment to be audio and video recorded. If you decide you would prefer not to have your appointment recorded, your appointment will not be affected in any way and you can still take part in the study. If your appointment is recorded (video and audio or just audio), you will be asked again after the consultation if you are still happy for the recording to be used as part of our research. You can say no at that point, and we will delete the recording. Additionally, immediately after the appointment, you may be asked to complete a short survey to find out about your experiences of the consultation. This survey will also be optional.

**What are the likely benefits and disadvantages of taking part?**

By taking part in the research, you will receive an estimate of your future risk of developing breast cancer. This information may help you to decide if you would like to make lifestyle changes to reduce that risk. If you are found to be at increased risk, you will also have the opportunity to discuss your results with your GP and if appropriate and with your agreement, will be offered a referral to specialist care to see if you are eligible for earlier screening or medication to reduce the risk of developing breast cancer. By taking part, you will also be contributing to research that could help patients and doctors in the future.

There are no medical risks in taking part. Still, learning about your risk of developing breast cancer in the future can potentially cause anxiety. To make sure this affects you as little as possible, we will provide you with information about local services and websites where you can find further information and support.

**Will my taking part in this study be kept confidential?**

Cambridge University Hospitals NHS Foundation Trust (CUHNFT) and the University of Cambridge are joint sponsors for this study based in the United Kingdom. CUHNFT and the University of Cambridge will be using information from you and your medical records in order to undertake this study and will act as joint data controllers. This means that both organisations are responsible for looking after your information and using it properly.

The University of Cambridge and CUHNFT will keep all identifiable information about you for 1 year after the study has finished. If you consent for us to use your data in future research, some personal data will be stored for longer than 1 year after the study has finished. Your rights to access, change or move your information are limited, as we need to manage your information in specific ways in order for the research to be reliable and accurate. If you withdraw from the study, we will keep the information about you that we have already obtained. To safeguard your rights, we will use the minimum personally-identifiable information possible. If you lose capacity after consenting to take part in this study, the research team will keep and make further use of identifiable data/tissue already collected.

You can find out more about how we use your information using the following links:

For Cambridge University Hospitals NHS Foundation Trust, please visit:

<https://www.cuh.nhs.uk/corporate-information/about-us/our-responsibilities/looking-after-your-information>, or email the Data Protection Officer at: [cuh.gdpr@nhs.net](mailto:cuh.gdpr@nhs.net)

For University of Cambridge, please visit: <https://www.medschl.cam.ac.uk/research/information-governance/>, or email  the Information Governance team at: [researchgovernance@medschl.cam.ac.uk](mailto:researchgovernance@medschl.cam.ac.uk)

[https://www.hra.nhs.uk/planning-and-improving-research/policies-standards-legislation/data-protection-and-information-governance/gdpr-guidance/templates/transparency-wording-for-all-sponsors/](https://eur03.safelinks.protection.outlook.com/?url=https%3A%2F%2Fwww.hra.nhs.uk%2Fplanning-and-improving-research%2Fpolicies-standards-legislation%2Fdata-protection-and-information-governance%2Fgdpr-guidance%2Ftemplates%2Ftransparency-wording-for-all-sponsors%2F&data=05%7C01%7Cfsd26%40universityofcambridgecloud.onmicrosoft.com%7Cff62dc967f2347c442ee08db680f1a18%7C49a50445bdfa4b79ade3547b4f3986e9%7C1%7C0%7C638218187189524539%7CUnknown%7CTWFpbGZsb3d8eyJWIjoiMC4wLjAwMDAiLCJQIjoiV2luMzIiLCJBTiI6Ik1haWwiLCJXVCI6Mn0%3D%7C3000%7C%7C%7C&sdata=thhIWFUwB7ZNUXqhzjaCjVJP3cwSfk%2BFG0gdcKRBjiM%3D&reserved=0)

With your consent, we would also like to store your personal details (for example your name and date of birth) for up to 10 years to link data from this study to NHS registries such as the National Cancer Registration and Analysis Service (NCRAS) and to the Hospital Episodes Statistics (HES) for use in future research. You will be asked if you agree to this on the consent form and can refuse while still taking part in this study.

With your consent, we would also like to store the DNA extracted from your saliva sample for potential use in new studies in the future. We would also like to be able to share your data and DNA sample with other researchers, which may be international and may include commercial partners. In a legal sense your sample will be treated as a “gift” and you will have no claim over the sample should the results of this research, or future studies, lead to commercial development. You will be asked if you agree to this on the consent form and can refuse while still taking part in this study.

Researchers from the CanRisk-GP study team will use your name and contact details to contact you about the research study, and make sure that relevant information about the study is recorded for your care, and to oversee the quality of the study. Individuals from the Research Governance office at the Cambridge University Hospitals NHS Foundation Trust and The University of Cambridge as well as regulatory organisations may look at your research records to check the accuracy of the research study. Researchers from the CanRisk-GP study team will pass these details to the Research Governance office at the Cambridge University Hospitals NHS Foundation Trust and The University of Cambridge along with the information collected from you. The only people in the Cambridge University Hospitals NHS Foundation Trust and University of Cambridge who will have access to information that identifies you will be people who need to audit the data collection process and the study researchers.

All information that is collected from you during the course of the study will be stored securely. The electronic data collected from questionnaires and the result of MyCanRisk app will be stored on a secure website. Data will be downloaded from the website for analysis where it will be stored on the University of Cambridge Secure Research Computing Platform (SRCP), which is registered under the School of Clinical Medicine’s NHS Digital Data Security and Protection Toolkit.

Audio recordings will be saved onto a password-protected computer. They will be uploaded to an external transcription company who will sign a confidentiality agreement. The recordings will then be uploaded to the University of Cambridge Secure Research Computing Platform (SRCP).

Data collected from video-recorded consultations will be transferred from the video recorders onto an encrypted hard drive and the original file deleted from the video recorder before leaving the general practice. The file will then be transferred onto the secure data hosting server.

Any data transferred will not contain personal identifiable information except for communications between the research team, YourGene (saliva sample and processing company) and GP surgeries and within the research team about specific participants and their visits, care or data. If you have agreed for us to use your data in future research, this data will be securely transferred to the new study at the end of this study.

If your responses to any of the questionnaires indicate that you are more worried about your health than other people, then one of the clinicians within the research team may contact you and inform your GP. The normal safeguarding procedures relating to notification of your identity as appropriate also still apply in the unlikely event that the research team consider that you or another person would be at risk of harm.

**How do I withdraw from the study?**

If you agree to take part, you can still change your mind later on. If you decide to withdraw from the study, we will keep the information about you that we have already obtained. Please contact the research team using the contact details at the bottom of this page to withdraw from the study. If you chose not to take part during an interview or the appointment with your GP you can interrupt it at any time. If the appointment is being recorded the GP will stop the recorder at any time if you wish.

**What if there is a problem?**

If you have any concern about any aspect of this study, please contact us (the research team) and we will do our best to answer your questions – our contact details can be found at the end of this information sheet.

If you are unhappy with a primary care service, such as your GP practice or pharmacist, you can complain either directly to the Practice Manager of the practice or if you prefer to NHS England, the organisation which manages complaints for these services:

- Telephone: [telephone number] (Monday to Friday 8am to 6pm, excluding bank holidays)
- Email: [email address]
- Write to: [post address]

If you are unhappy with a Cambridge University Hospitals NHS Foundation Trust service and wish to complain formally or have any concerns about any aspect of the way you have been approached or treated during this study, you can do this through the NHS complaints procedure. In the first instance it may be helpful to contact the Patient Advice and Liaison Service (PALS) at your hospital.

Patient Liaison and Advice Service (PALS) Contact Details:

- Telephone: [telephone number]
- Email: [email address]

**Who is organising this study?**

The study is organised by Cambridge University Hospitals NHS Foundation Trust and the University of Cambridge.

Cambridge University Hospitals NHS Foundation Trust, as a member of the NHS Clinical Negligence Scheme for Trusts, will accept full financial liability for harm caused to participants in the study caused through the negligence of its employees and honorary contract holders. There are no specific arrangements for compensation should a participant be harmed through participation in the study, but no-one has acted negligently.

The University of Cambridge will arrange insurance for negligent harm caused as a result of protocol design and for non-negligent harm arising through participation in the study.

**Who has approved the study?**

All research in the NHS is reviewed by an independent group of people, called a Research Ethics Committee (REC), which is there to protect your safety, rights, wellbeing, and dignity. This study has been reviewed and given favourable opinion by East of England – Cambridge Central REC and governance approval by the Health Research Authority.

**What will happen to the results of the study?**

The results of the study will be used to improve MyCanRisk and to inform future research to include CanRisk within general practice. We will also present the findings at scientific meetings and in scientific journals and send a summary of the results to everyone who takes part in the study. Quotations from the interview data will be anonymised and any other identifying information removed in these publications.

At the end of the study the data from the questionnaires, MyCanRisk and medical records will be anonymised to remove any names or personal details and become “open data”. This means that it will be stored in an online database so that it is publicly available. This process is integral to the research process as it allows other researchers to verify results and avoid duplicating research. Anonymised data will be made available on a website, free of charge, to anyone interested in the research, or who wishes to conduct their own analysis of the data. We would therefore have no control over how these data are used. We will remove any names or personal details though that might allow you to be identified.

If you take part in one of the optional interviews or agree to your consultation with a GP being audio recorded, all names and places will be removed from transcripts. We might make transcripts available to other academic researchers in the future.

**Contact us**

If you are interested in taking part in the study or would like some further information, then please contact the CanRisk-GP study team.

**Phone:**  [telephone number] (Monday to Friday 9.30 am – 5 pm)

**Email:**  [email address]

If you have any concerns about the way you have been approached or treated, please contact the Chief Investigator, Dr Juliet Usher-Smith on [jau20@medschl.cam.ac.uk](mailto:jau20@medschl.cam.ac.uk) or write to her at

Dr Juliet Usher-Smith

The Primary Care Unit, Department of Public Health and Primary Care

East Forvie Building, University of Cambridge, CB2 0SR

**Thank you very much for reading this information sheet and considering taking part in this study**

*Go to the study website using the link in your letter of invitation if you would like to take part or to tell us why you don’t wish to or can’t take part.*
