# Supplementary material for: CanRisk-GP protocol: A feasibility study of incorporating proactive multifactorial breast cancer risk assessment into general practice
Source: PLoS One. 2025 Nov 26;20(11):e0336902. doi: 10.1371/journal.pone.0336902 (PMC12654911; doi:10.1371/journal.pone.0336902)
Supplement: S3 File — (PDF) [file pone.0336902.s003.pdf]

CanRisk-GP: a feasibility study of incorporating proactive multifactorial breast cancer risk assessment into general practice

Research Team

Chief Investigator:	Dr Juliet Usher-Smith, Assistant Professor of General Practice
Co-Investigators:	Professor Fiona Walter, Professor of Primary Care Research<br>Professor Steve Morris, RAND Professor of Health Services Research<br>Professor Jon Emery, Herman Chair of Primary Care Cancer Research<br>Professor Antonis Antoniou, Professor of Cancer Risk Prediction<br>Professor Marc Tischkowitz, Professor of Medical Genetics<br>Professor Doug Easton, Professor of Genetic Epidemiology<br>Dr Stephanie Archer, Senior Research Associate<br>Dr Francisca Stutzin Donoso, Research Associate<br>Adam Stokes, Project Manager<br>James Brimicombe, Senior IT Specialist / Data Manager<br>Cameron Wilson, PhD student<br>Laura Stylianou, Study manager<br>Amy Lafont, Study co-ordinator
Collaborators:	Dr Timothy Carver, Computer Programmer<br>Dr Penelope Moyle, Consultant Radiologist<br>Dr Kathryn Taylor, Director of Breast Screening and Consultant Radiographer<br>Beverley Speight, Principal Genetic Counsellor
PPIE representatives:	CanRisk Patient Public Partners Group

Funder

This study is funded by Cancer Research UK (PPRPGM-Nov20\100002).
Juliet Usher-Smith is funded by an Advanced Fellowship from the National Institute for Health and Social Care Research (NIHR300861). Jon Emery is supported by an NHMRC Investigator grant (APP1195302). The University of Cambridge has received salary support for Douglas F Easton from the NHS in the East of England through the Clinical Academic Reserve.

Sponsor

University of Cambridge and Cambridge University Hospitals NHS Foundation Trust

IRAS Ref: 326051
REC Ref: 23/EE/0199
Trial Registry (ISCRTN): ISRCTN17376192

Contents

1. Background and Rationale 4
2. Objectives 4
3. Methods and Procedures 5
3.1 Study design 5
3.2 Study setting 5
3.3 Practice recruitment 5
3.4 Participant recruitment, invitation process and consent 5

3.5 Breast cancer risk assessment 8
3.6 Return of results to participants and subsequent management 9
3.7. Outcomes 14
3.8 Data collection 15
3.8.1. Baseline data collection 15
3.8.2 Follow-up data 15
3.8.3 Process evaluation and qualitative data collection 16
3.9 Withdrawal of consent and death 19
3.10 Safety Reporting 19
3.11 Sample size 20
3.12 Analysis 21
3.12.1 Quantitative data analysis 21
Uptake of breast cancer risk assessment 21
The quantity of self-report data using MyCanRisk 22
Psychological and behavioural impact of multifactorial breast cancer risk assessment 22
3.12.2 Process evaluation and qualitative data analysis 22
4. Data Management 22
4.1 Data storage and access 22
4.2 Data anonymisation 24
5. Study management 25
5.1 Participating Sites 25
5.2 Site Training 26
5.3 Sponsor 26
5.4 Funding 26
5.5 Portfolio Adoption 26
5.6 Peer Review 26
5.7 Public participation in the research 26
5.8 Risk Assessment 27
5.9 Oversight Committees 27
5.10 Protocol Deviations and Incidents 27
5.10.1 Protocol Deviations 27
5.10.2 Incidents 27
5.10.3 Protocol Violation 27
5.11 Monitoring 28
5.12 Trial Master File 28
5.13 Central Delegation of Duties and Training 28
5.14 Dissemination 28
5.15 Data sharing 28

5.16 Ethical review and reports 28
5.17 Insurance and Indemnity 29
5.18 Archiving 29
7. References 29
8. Protocol Appendices 32
8.1 Appendix 1: Document Appendices 32
8.2 Appendix 2: Summary of Study Activities 33
8.3 Appendix 3: Glossary of Key Terms 33
8.4 Appendix 4: Summary of Changes 34

1. Background and Rationale

The National Institute for Health and Care Excellence (NICE) recommends that women at moderate and high risk of breast cancer be offered additional breast screening and risk reducing medication from age 30[1]. Much research is being done to identify women over 50 years old who are at above-population level risk through the national breast cancer screening programme[2,3]. Women under 50 years though need to be referred by GPs into Clinical Genetics Services. Currently this relies on women self-presenting to GP practices with concerns about their breast cancer risk. Research has shown that this opportunistic approach leads to only a small fraction of those at moderate or high risk being identified: in one survey among screening attendees, it was estimated that 8.8% of women in their forties would be eligible for additional screening/surveillance and risk-reducing medication[4], but only 17.5% of that group had been seen in family history or clinical genetics services. Relying on women to self-present with concerns may also exacerbate existing health inequalities or disadvantage groups who have lower levels of health literacy.

In June 2022, following a stakeholder consultation, the NICE statement recommending identification of women only when they present with concerns was withdrawn to allow the proactive identification of women at increased risk of breast cancer within primary care. This shift to proactive identification substantially changes the role of primary care, in effect paving the way for a primary care-based screening programme to identify those at moderate or high risk of breast cancer.

We have conducted a review of the literature surrounding proactive breast cancer risk assessment within primary care against the consolidated framework for screening[5]. In that review we found that proactive risk assessment for women under 50 years currently satisfies many of the standard principles for screening. Most notably, there are large numbers of women at moderate or high risk currently unidentified, risk models exist that can identify those women with reasonable accuracy, and management options that offer the opportunity to reduce breast cancer incidence and mortality in that group. However, there remain a number of uncertainties and research gaps, particularly around uptake of the risk assessment and subsequent risk-reducing options, how risk is communicated, and how best to integrate proactive breast cancer risk assessment within primary care, that need to be addressed before these benefits can be realised.

2. Objectives

The specific objectives of this feasibility study are:

1. To quantify uptake of proactive multifactorial breast cancer risk assessment using CanRisk in general practice amongst women under 50;
2. To assess the acceptability of all stages of proactive multifactorial breast cancer risk assessment using CanRisk within general practice from the perspective of women and GPs;
3. To compare the number of women identified as at moderate or high risk of breast cancer following proactive multifactorial breast cancer risk assessment with those identified through routine care and the number expected from population-based studies;
4. To quantify the psychological and behavioural impact of proactive multifactorial breast cancer risk assessment using CanRisk in general practice amongst women;
5. To quantify the amount of data collected via the MyCanRisk public-facing data collection tool within a primary care population;
6. To estimate the distribution of breast cancer risk amongst women under age 50 taking up the opportunity for multifactorial risk assessment following proactive invitation and uptake of risk-reducing interventions amongst those at moderate or high risk to inform sample size calculations for the design of a future randomised controlled trial;

7. To estimate differences in uptake of proactive multifactorial breast cancer risk assessment and breast cancer risk distribution of women between general practices to inform the design and sample size calculations for a future cluster-randomised controlled trial;
8. To collect data to enable estimation of the cost of delivering proactive multifactorial breast cancer risk assessment using CanRisk within general practice.

3. Methods and Procedures

3.1 Study design

A pragmatic multicentre feasibility study of proactive multifactorial breast cancer risk assessment within general practice (GP).

3.2 Study setting

The study will take place within 5 to 8 GP practices in the East of England.

3.3 Practice recruitment

GP practice recruitment will be supported by the Clinical Research Network (CRN) Eastern who will approach practices across the East of England who refer all patients with a family history of breast cancer to Cambridge University Hospital NHS Foundation Trust. Practices will be recruited from different areas to maximise participant socio-demographic and ethnic diversity.

Further information on participating site requirements are outlined in section 5.1.

3.4 Participant recruitment, invitation process and consent

Participant recruitment and flow through the study is summarised in Figure 1.

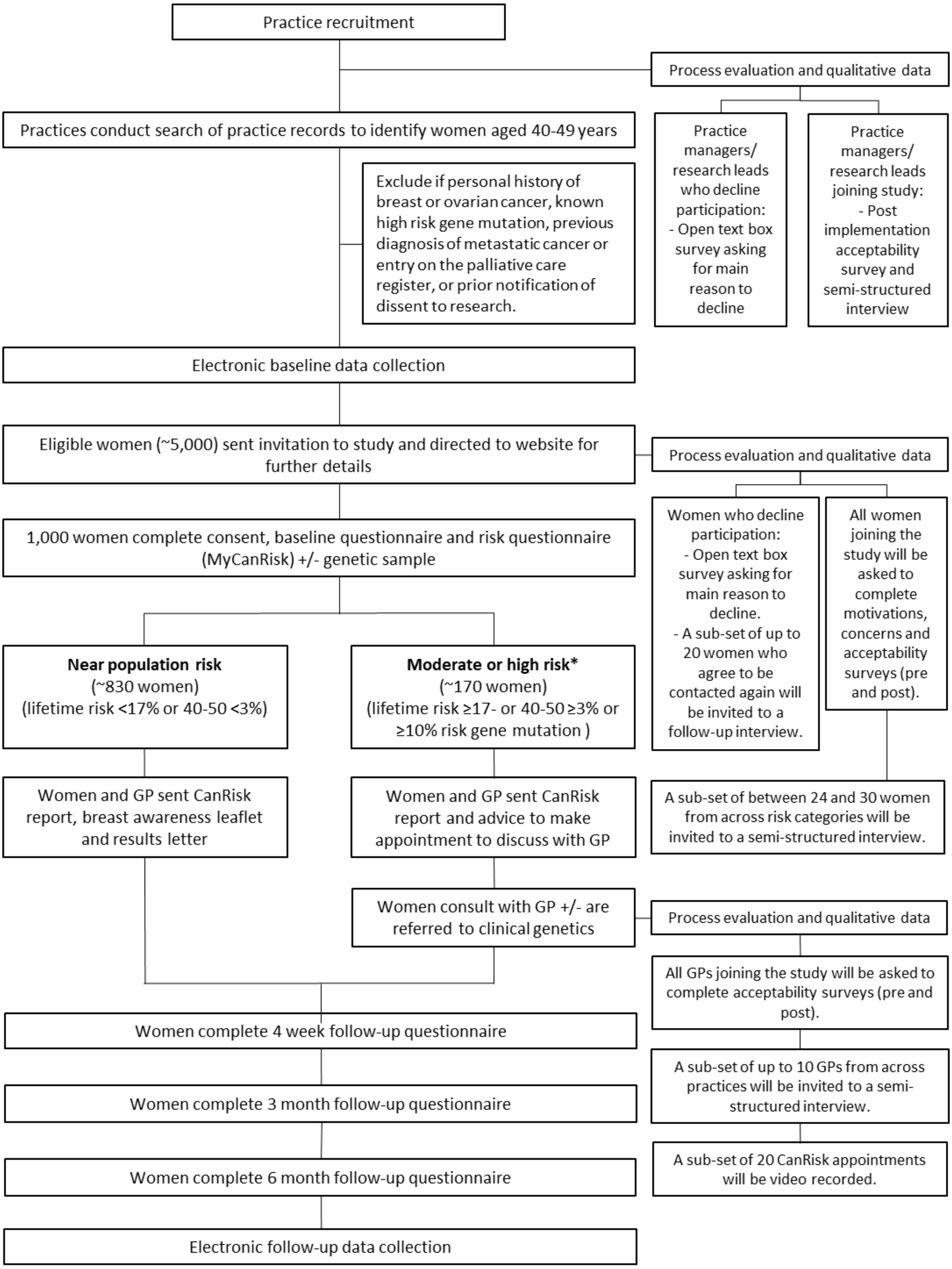

* Or at least 1 FDR or SDR with BC and any of:

- Sarcoma in relative < 45 years
- Glioma or childhood adrenal cortical carcinomas
- Complicated patterns of multiple cancers at a young age
- Jewish ancestry

Figure 1. Study design and participant flow

Inclusion criteria:
Women will initially be eligible for the study if they are:

- aged 40-49 years, and
- registered with a participating general practice, and
- capacity to consent, and
- English speaker.

The upper limit of age of 49 years is chosen because all women are invited to participate in the national breast cancer screening programme at age 50 and there is considerable work being conducted to evaluate the potential for offering risk assessment alongside mammography to identify women attending screening who are above near population risk [2,3,6]. The principal target population for proactive identification in primary care is, therefore, those women under 50 who would potentially benefit from interventions but are not yet eligible to be invited for population-based screening. An upper age limit of 48 has been implemented in the initial search, to prevent including women who may otherwise reach the age of 50 before completing the intervention.

The initial lower limit of age 40 years is chosen because the main intervention amongst women at moderate and high risk of breast cancer is enhanced breast screening and age 40 is the minimum age in Cambridgeshire and Peterborough at which women at moderate risk are eligible for this. Women identified as being at moderate risk younger than age 40 are advised to return for a repeat assessment at age 40. Risk-reducing medication is offered to women who are at moderate risk from age 35 years. However, uptake of risk-reducing medication at that age has been shown to be extremely low amongst women identified through genetics clinics [7]. We have, therefore, chosen initially to include only women aged 40 or over so that all women identified as being at moderate risk through this study will be immediately eligible for enhanced breast screening. We will consider reducing this minimum age to 35 years if recruitment into the study is less than expected.

Exclusion criteria:
Women will be excluded if they have:

- a personal history of breast or ovarian cancer, or
- a known high risk gene mutation, or
- a previous diagnosis of metastatic cancer or entry on the palliative care register, or
- prior notification of dissent to research, or
- lack of capacity to consent, or
- Non-English speaker.

Eligible women will be identified through searches of the electronic health records at General Practices. The searches have been developed alongside the CRN Eastern IT Specialist and will be conducted by members of staff within each General Practice. Depending on the population size of the practices taking part in the study, initially either all or a random sample of those women who meet the eligibility criteria will be invited to take part in the study. If uptake rates are very different between socio-demographic groups (i.e. age, Index of Multiple Deprivation (IMD) or ethnicity) then we may consider oversampling women within those groups with lower uptake. Eligible women will be sent an invitation letter (Appendix 2 – Invitation letter to women) and participant information sheet (PIS) (Appendix 3 – Participant information sheet) by post from their GP practice. Two to four weeks later, women who have not completed the consent form and/or have not made contact with the research team will be sent a reminder letter by post from their GP practice (Appendix 4 – Reminder letter). The wording of the invitation letters has been informed by behavioural studies and reviewed by our Patient and Public Involvement/Engagement (PPIE) partners.

Going to the link or QR code included in the invitation or reminder letters will take women to a study website, hosted on Qualtrics, where those women wishing to take part will then complete an online consent form (Appendix 5 – Participant consent form). Those who do not wish to take part can follow the same link to provide information on their reasons if they wish to and be offered the opportunity to be interviewed to provide further information (Appendix 22 – Decliner form with PIS and ICF for interviews) The contact details of the research team will be provided in the invitation letter, allowing women the opportunity to ask questions before giving consent. The completed online consent form will be sent automatically to the participant by email and to the research team.

Further exclusion criteria:
Women will be asked further screening questions on the consent form. The following women will be excluded:

- Those who are aware of a high-risk breast cancer gene already identified within their family.
- Those who are no longer with a participating GP.
- Those who have a history of breast or ovarian cancer.

Whilst potential participants identified in the electronic health record search would not initially meet the latter two exclusion criteria, there is a possibility that a patient may meet them by the time they consent to the study. Patients who meet these exclusion criteria will be notified that they are not able to continue with the study.

Those who are excluded due to a high-risk breast cancer gene within their family will be advised that they should make an appointment to discuss their breast cancer risk with their GP and will not be able to continue with the study consent form.

After providing online consent, all participants will complete a short baseline questionnaire (Appendix 6 - Baseline questionnaire). Participants who do not complete the baseline questionnaire within two weeks of completing their consent form will receive a reminder. Contact details of the research team will be provided on the study website and in the invitation letters if women need help completing the questionnaire. The baseline questionnaire will be hosted on Qualtrics. On completion of the baseline questionnaire, all other participants will then be given the opportunity to provide data for a breast cancer risk assessment (see section 3.5). Separate consent will be obtained for the process evaluation (see section 3.8.2).

Recruitment will continue for 6 months or until we have recruited 1,000 women.

3.5 Breast cancer risk assessment

Personalised risk estimates will be generated using the CanRisk tool. CanRisk is a multifactorial-cancer-risk-prediction tool incorporating the BOADICEA risk model that combines genetic, family history, lifestyle and hormonal risk factors to calculate the future risk of developing breast cancer, as well as the risk of being a carrier of a pathogenic variant (PV). Population-based studies of BOADICEA in pre-menopausal women or those under the age of 50 have shown that this has moderate-to-good discrimination with AUCs or c-indices of 0.68-0.69[8,9] and is well calibrated with a ratio of expected to observed number of cases (E/O) of 0.97[9].

For this study, personalised risk estimates will be calculated based on family history, demographic, lifestyle and hormonal factors, in addition to common genetic susceptibility variants in the form of a polygenic risk score (PGS). Data on family history, lifestyle and hormonal factors will be collected via the MyCanRisk app (Appendix 7 – screenshots from MyCanRisk). Women will be sent a link by the research team to the MyCanRisk app after completing the consent and baseline questionnaire. Participants who do not complete their MyCanRisk assessment within 2 weeks of receiving their link

will receive a reminder. CanRisk and MyCanRisk is maintained by the CanRisk team and is hosted on the Cambridge University Information Service (UIS). UIS is ISO27001 accredited and NHS Security and Protection Toolkit compliant.

MyCanRisk is a Progressive Web App that supports the on- and off-line collection of personal risk factor information (including information on lifestyle and women's health) alongside the detailed family history required to conduct a CanRisk calculation. Users have the opportunity to check the information added to MyCanRisk before sending the encrypted data to their healthcare professional (or the research team, in this instance), where data can be added/updated (e.g. the inclusion of the PGS) before the CanRisk calculation is completed.

Common genetic susceptibility variants will be assessed from a saliva sample using PGS testing. Our current external providers are Yourgene and Allelica, but other similar providers, both internal (a University of Cambridge or a Cambridge University Hospitals NHS Foundation Trust Department) or external (commercial) providers may be used for sample processing and PGS analysis in future if needed. Women will be posted a saliva sampling kit by the research team. The saliva sampling kit will include a covering letter (Appendix 8 – saliva sample kit covering letter) with detailed instructions on how to take the sample and a pre-paid return envelope to return the kit to the research team. Participants will be asked to include a participant information slip with their sample to confirm their name and month/year of birth to ensure the sample is linked to the correct participant. Upon receipt, the details will be verified, and the form will be confidentially destroyed. Participants will have the option not to have PGS testing. Participants who do not return their saliva sample kit within three weeks of it being sent to them will receive a reminder. The returned saliva sampling kit will be processed by the research team and sent to Yourgene Genomic Services, located at Skelton House, Lloyd Street North, Manchester Science Park, Manchester M15 6SH (or other suitable provider). Yourgene (or other suitable provider) will extract DNA from the samples, perform quality control processes and prepare the sample for genomic analysis.

Samples which pass quality control will be run using microarray technology, specifically the Illumina Global Screening v4 Array (GSAv4). The output from the GSAv4 analysis (IDAT files) will be transferred to Allelica (or other suitable provider) through a secure file transfer protocol (SFTP) connection. Allelica (or other suitable provider) will run the IDAT files to calculate a polygenic risk score (PGS) for breast cancer risk. PGS results will either be returned to Yourgene (or other suitable provider) to then send to us, or delivered directly to us, depending on which is more appropriate. These data will be used in the preparation of a report to be delivered back to women who participated in the study via the research team.

The PGS result will aim to be returned to the research team within approximately 4 weeks of YourGene (or other suitable provider) receiving the samples. This result will be combined with the data on family history, lifestyle and hormonal factors provided by the participants via MyCanRisk by the research team and a personalised risk estimate generated using the CanRisk tool. For those women opting not to have PGS testing, the personalised risk estimate will be based on family history, lifestyle and hormonal factors only.

3.6 Return of results to participants and subsequent management

The process for returning results to participants and subsequent management is shown in Figure 2.

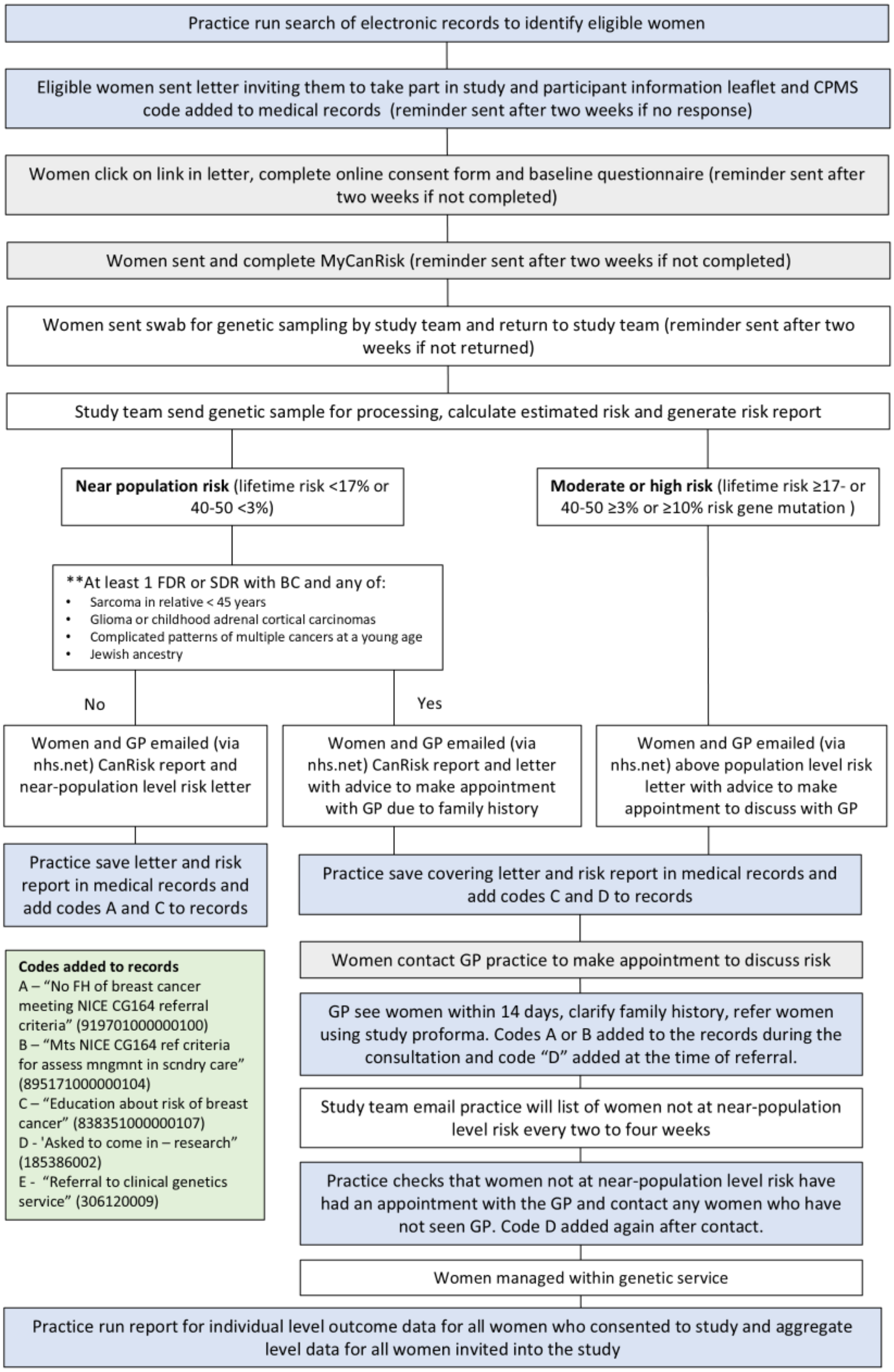

Figure 2. Process for estimation of risk, communication of risk to participants and GPs and subsequent management. Blue shading indicates activities completed by general practice staff.

Based on the CanRisk estimate and current NICE guidelines[1], participants will be categorised into one of three risk groups: (i) near population level risk, (ii) near population level risk but with additional features in the family history, and (iii) moderate or high risk (Table 1).

Table 1. Risk categorisation

Risk	Near population risk	Moderate risk	High risk
Lifetime risk from age 20	<17%*	17-30%	≥30%
Risk between ages 40 and 50	<3%*	3-8%	≥8%

* Without any of the following features within the family history: sarcoma in relative < 45 years; glioma or childhood adrenal cortical carcinomas; complicated patterns of multiple cancers at a young age; Jewish ancestry.

Women with an estimated risk in the near population level risk group and without any additional risk factors within their family history (see Table 1) will be sent a letter by email from the research team informing them that they are at near population level risk for breast cancer (Appendix 9- Near population risk results letter) along with a breast awareness leaflet (Appendix 10 – Breast awareness leaflet) and the risk output from the CanRisk tool (Appendix 11 - Example of output from CanRisk tool). In line with current NICE guidance, the breast awareness leaflet includes details about the woman's risk as well as breast awareness information, lifestyle advice regarding breast cancer risk, including information about Hormone Replacement Therapy (HRT) and oral contraceptive use, diet and alcohol, breastfeeding, family size and timing, contact details of local and national support groups and advice to return to discuss any implications if there is a change in family history or breast symptoms develop. The output from the CanRisk tool contains the woman's risk of developing breast cancer in the next 5 years, 10 years and between now and the age of 80. It also presents the woman's risk category according to NICE (CG164), her risk of developing breast cancer throughout her lifetime relative to the UK population, and her risk of carrying a genetic PV that is relevant to her risk of developing breast cancer in the future. Additional information includes the woman's family pedigree, a summary of cancer diagnoses in her family, a summary of the information included in the breast cancer model used to calculate her risk and the woman's breast cancer polygenic score. The format and presentation of that output has been developed following recommendations from experts in breast cancer risk prediction working in primary care and clinical genetics. A copy of both the letter and risk output will be sent to the participants' GP. The GP (or a delegated individual within the practice) will add the codes "*No FH of breast cancer meeting NICE CG164 referral criteria*" and "*Education about risk of breast cancer*" to the medical records.

Women with an estimated risk at near population level but with additional risk factors within their family history (see Table 1) will be sent the risk output from the CanRisk tool and breast awareness leaflet along with a letter from the research team informing them that some of the features of their family history need clarifying and may mean that the estimated risk is inaccurate and advising them to make an appointment to discuss with their GP (Appendix 12 - Near population risk with additional family history risk factors results letter). A copy of the letter and the risk output from the CanRisk tool will also be sent to the participants' GP. The GP (or a delegated individual within the practice) will add the codes "*Education about risk of breast cancer*" and "*Asked to come in – research"* to the medical records.

Women with an estimated risk in the moderate or high risk groups will be sent the risk output from the CanRisk tool and breast awareness leaflet along with a letter from the research team informing them that they may be at above average population level risk for breast cancer and advising them to make an appointment to discuss with their GP (Appendix 13 - Moderate and high risk results letter). A copy of the letter and the risk output from the CanRisk tool will also be sent to the participants' GP. The GP (or a delegated individual within the practice) will add the codes "*Education about risk of*

breast cancer" and "*Asked to come in – research"* to the medical records. If during the consultation with the GP it becomes clear that a woman has added incorrect information to the MyCanRisk app, GPs will be advised to ask the woman to contact the research team to arrange for their risk factor information to be updated and a new risk output will then be sent to both the woman and the GP by the research team. In all other cases, the GP (or a delegated individual within the practice) will add the code "*Mts NICE CG164 ref criteria for assess mngmnt in scndry care*" to the medical records.

Women confirmed to have additional risk factors within their family history or at estimated moderate or high-risk for breast cancer will be offered referral to the regional clinical genetics service. A referral proforma will be provided for GPs to use for this purpose (Appendix 15 – Referral proforma for GPs). Women referred to the regional clinical genetics service will then be managed in line with current practice (Figure 3), with those women confirmed to be at moderate or high risk being offered risk-reducing medication and enhanced screening in line with NICE guidelines[1]. The only change to their care will be that women referred as part of this study will not need to complete a separate family history questionnaire (usually sent to women after a "Reject for further information" triage decision) as this data will already have been collected from study participants in the MyCanRisk app.

To ensure all women estimated to be at population-level risk but with additional risk factors and those at moderate or high risk have received the recommendation to arrange a consultation with their GP and have been given sufficient opportunity to make that consultation, processes will be implemented within each General Practice to ensure that women contacting the practice having received their results letter are offered a consultation within 14 days. The precise details of the process will be agreed with each General Practice but it is anticipated that one or more GPs will be lead GPs for this study in each practice. Guidance for receptionists in each practice will be generated to support the booking process. Additionally, each practice will review the medical records every 2 to 4 weeks for women who were identified at population-level risk with additional risk factors or moderate or high risk and check if those women have attended for a consultation with a GP. Any women who have not booked a consultation or already consulted with a GP will be contacted by the practice administrative team and supported to book an appointment if they wish to. Patients declining a consultation after contact from the practice will not be contacted further. Each patient contact and any reasons for not wishing to consult with a GP will be recorded by the practice staff within the patient electronic health record.

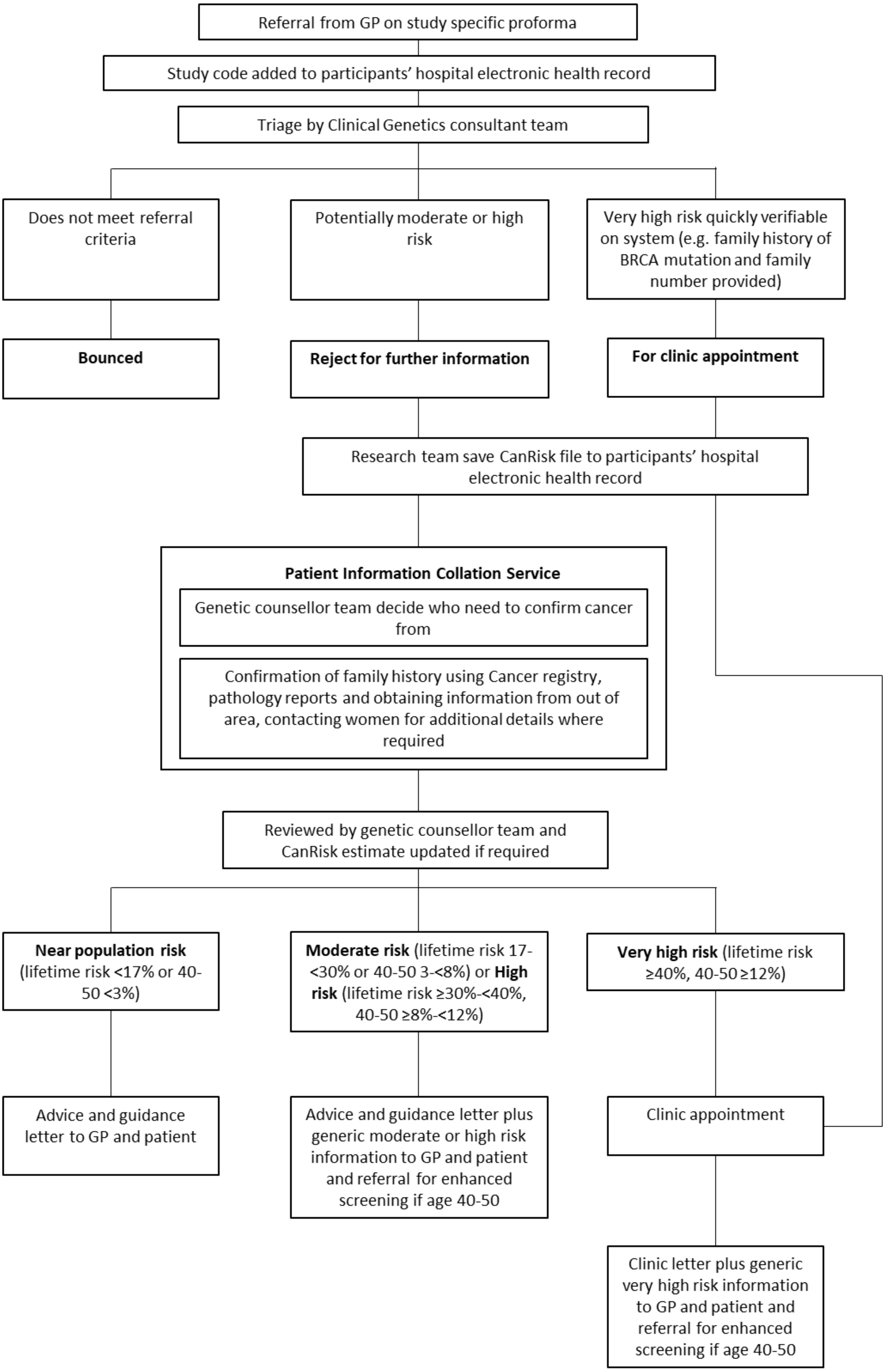

Figure 3. Pathway for women once referred to clinical genetics

3.7. Outcomes

The study outcomes are:

- Uptake of multifactorial breast cancer risk assessment
    - The proportion of eligible women invited in each practice who consent to take part
    - The socio-demographic distribution of responders vs non-responders in each practice
    - The proportion of eligible women invited in each practice who consent to complete the full risk assessment including PGS
    - The proportion of eligible women invited in each practice who consent to complete the risk assessment but decline the PGS
- Distribution of breast cancer risk
    - The proportion of women who consent who are at moderate or high risk on initial CanRisk assessment in general practice
    - The proportion of women who consent who are at moderate or high risk following assessment in secondary/tertiary care
- Identification of women at moderate and high risk of breast cancer
    - The proportion of registered women aged 40-49 years at participating practices who are at moderate or high risk of breast cancer after assessment in secondary/tertiary care compared with the proportion of women aged 40-49 years at moderate or high risk of breast cancer after referral to secondary/tertiary care at non-participating practices across Cambridgeshire and Peterborough during the same time period and compared with the proportion of registered women aged 40-49 years at participating practices in the 12 months prior to the study
- Uptake of risk-reducing interventions
    - The proportion of those at moderate or high risk who attend for a GP appointment
    - The proportion of those at moderate or high risk who are referred to secondary/tertiary care
    - The proportion of those at moderate or high risk who take up enhanced screening and/or preventative medication
- The amount of information collected through MyCanRisk
    - The presence of information completed for non-family history risk factors (e.g. height, weight, alcohol, women's health) obtained through MyCanRisk
    - The number of family members included in the pedigree collected through MyCanRisk
- Psychological and behavioural impact of multifactorial breast cancer risk assessment
    - The change in psychological and behaviour measures following multifactorial breast cancer risk assessment in those at near-population level risk, those at moderate risk and those at high risk at four weeks, three months and six months after receiving the risk estimate
- Acceptability
    - The acceptability of the pathway of proactive multifactorial risk assessment within primary care to women
    - Response rates and completion of questionnaires
    - The willingness for practices to take part
    - The acceptability of proactive multifactorial risk assessment within primary care to GPs
- Costs
    - Time and workload impact on GP practices
    - Healthcare utilisation

Together with data on the acceptability of the proactive multifactorial breast cancer risk assessment, the following progression criteria will be evaluated in order to judge whether a subsequent full trial of proactive multifactorial breast cancer risk assessment will be feasible: 20% uptake into the study; 60% response to follow-up questionnaires; 80% of women receiving their breast cancer risk and offered subsequent risk management advice/referral. If any of those criteria are not met, we will use the data from the process evaluation to adapt the approach for subsequent feasibility and/or pilot studies before progression to a subsequent full trial.

3.8 Data collection

3.8.1. Baseline data collection

Participant baseline data will be collected via online questionnaires (Appendix 6 – Baseline Questionnaire). The questionnaires will include socio-demographic questions alongside validated measures of numeracy (Schwarz numeracy scale [10]), perceived risk of breast cancer, cancer worry (Lerman cancer worry scale-R[11]), anxiety (short form of the State-Trait Anxiety Inventory (STAI)[12]), quality of life (EQ-5D-5L[13]) and intolerance of uncertainty (short version of the Intolerance of Uncertainty scale[14]), intention to take up breast cancer screening in the future, physical activity, time orientation (the Zimbardo Time Perspective Inventory[15]), health literacy[16], beliefs about breast cancer[17], the multidimensional impact of cancer risk assessment (MICRA)[18] and healthcare utilisation related to breast health in the previous 6 months (Table 2).

3.8.2 Follow-up data

Participant level follow-up data will be collected via participant questionnaires and from the GP electronic medical records for all individuals who have consented to participate in the study. Participants will be invited, by the research team, to complete questionnaires at 4 weeks and at 3 and 6 months after receiving their results letter (Appendices 16-18). These questionnaires will collect data from all participants on risk perception, psychological measures, including cancer worry, anxiety and quality of life, and lifestyle and behavioural outcomes, including healthcare utilisation, as well as data on uptake of risk management options and knowledge and attitudes towards these options amongst those women identified as at above population risk (Table 2). The research team will also collect data from the coded GP electronic medical records on consultations related to breast symptoms or breast cancer risk and, for those at above population risk, uptake of risk-reducing interventions. Data transferred from practices to research staff will be sent from NHS email to the study NHS email account and stored in a secure University of Cambridge location.

Practice-level aggregated data will also be extracted from the GP electronic medical records on the age, ethnicity and IMD of all eligible women. Practice-level and aggregated individual level data on the number of referrals of women potentially at above population-level risk for breast cancer and the outcomes of those referrals for all practices across Cambridge and Peterborough for the period of the study and for the practices participating in this study, for the 12 months prior to the study will be extracted from the electronic health records at the genetics clinic at Cambridge University Hospitals NHS Foundation Trust. These data will be extracted by members of the clinical team or those with honorary clinical contracts within Cambridge University Hospitals NHS Foundation Trust and transferred via NHS email. Data on the number of women registered at each practice will be extracted from publicly available National General Practice profiles (https://fingertips.phe.org.uk/profile/general-practice).

Table 2.

Construct	Baseline	Within 4 weeks	3 months	6 months
**Socio-demographic**				

Education status	•			
Postcode	•			
Family history				
Sarcoma in relative <45 years	•			
Glioma or childhood adrenal cortical carcinoma	•			
Multiple cancers in family at a young age	•			
Jewish ancestry	•			
**Outcomes**				
Risk perception				
Breast cancer risk perception (absolute)	•	•	•	•
Breast cancer risk perception (comparative)	•	•	•	•
Breast cancer risk conviction	•	•	•	•
Recall of risk information		•	•	•
Psychological				
Anxiety [12]	•	•	•	•
Cancer-specific worry [19]	•	•	•	•
Quality of life [13]	•	•	•	•
Impact of cancer risk assessment [18]		•	•	•
Satisfaction with test		•		
Views on the risk information[20]		•		
Attitude towards risk management options		•	•	•
Lifestyle and behavioural				
Current behaviour (breast self-examination)	•	•	•	•
Intention to attend screening	•	•	•	•
Change in behaviour (weight, alcohol consumption, exercise)		•	•	•
Healthcare utilisation	•			•
Actions following risk assessment*				
Consultation with GP (or intention if not done)		•	•	•
Uptake of risk reduction strategies				
Enhanced screening		•	•	•
Risk reducing medication		•	•	•
Surgery		•	•	•
Process evaluation**				
Acceptability	•		•	
Motivations and concerns	•			
**Moderators**				
Numeracy [10]	•			
Health literacy[21]	•			
Beliefs about breast cancer[17]	•			
Time orientation [15]	•			
Intolerance of uncertainty [14]	•			

* Only for those at moderate/high risk and those at population risk with additional risk factors.
** As specified in section 3.8.2

3.8.3 Process evaluation and qualitative data collection

A mixed-methods process evaluation will be conducted to explore how proactive breast cancer risk assessment is delivered and perceived at practice and patient levels. The main four questions guiding this element of the study will focus on: 1) whether this feasibility study did what it was set out to do; 2) whether the different elements in the study design worked and why; 3) what the different stakeholders thought about CanRisk and how it was implemented in primary care; and 4)

whether implementing CanRisk in primary care is acceptable. We will collect data from women undergoing the study pathway, GPs leading the consultations with women at increased risk of developing breast cancer and practice managers and/or research leads from the different research sites.

The baseline questionnaire for all women who agree to take part will include questions covering their motivations and concerns regarding their participation in the study and the dimensions in the Theoretical Framework of Acceptability (TFA) [22] (Appendix 6 – Baseline questionnaire). The three month questionnaire will additionally include questions covering the dimensions in the TFA (Appendix 17 – Three month follow-up questionnaire).

We will aim to recruit up to 50 women to take part in semi-structured interviews, which will be conducted either face to face or online depending on the feasibility and interviewees' preferences. The interviews will be made up of three sub-sets of participants:

1. Those who complete the risk assessment
2. Those who decline or do not complete specific aspects of the study, including those who do not complete the risk assessment
3. Those who decline participation in the study

For sub-sets one and two, the interviews will cover their views on parts or all of the pathway (Appendix 19 – Participant information sheet for interviews with women; Appendix 20 – Interview schedule for women). We aim to interview women who have declined different components of the study and those at different risk categories to gain insight on the acceptability of the intervention for women across risk groups. Participants will only be contacted about an interview if they consented to be contacted in their initial consent form. Women may also be invited for an optional follow-up interview up to six months after the initial interview to gain more in depth understanding of later aspects of the pathway. Separate written consent will be taken prior to the interviews (Appendix 21 – Consent form for interviews with women; Appendix 21b – Consent form for FU interviews with women)
Additionally, participants who agree to take part in the study but decline PGS testing will be asked to provide a short description of their main reason(s) to decline PGS testing (Appendix 24 – PGS decliner form).

For the third sub-set, women who decline to participate in the study, the interviews will aim to shed light on potential barriers for future uptake (Appendix 23– Interview schedule for women who decline). Women who do not wish to take part in the study are advised to decline through the consent form, selecting they do not wish to participate in the study. Subsequently,
they will be asked to provide a short description of their main reason(s) not to join the study and whether they would like to participate in a semi-structured interview (Appendix 22 – Decliner form). Those signifying that they are interested in the interview will see the participant information sheet (Appendix 19 - Participant information sheet for Interviews with women) and consent form (Appendix 21 - Consent form for interviews with women participants).

All primary care clinicians taking part in this study will be invited to complete an electronic questionnaire after the study. The questionnaire will include questions around acceptability from the TFA and from the NoMAD checklist about the potential for the intervention to be incorporated into practice according to Normalisation Process Theory (NPT)[23] (Appendix 25 – Healthcare professional post-study questionnaire).Completion of the questionnaire will be taken as presumed consent for this component of the study, since all primary care clinicians asked to complete the

questionnaire will already have agreed to take part in the study and the questionnaires are anonymous. A sub-set of up to 10 primary care clinicians from across all practices will also be invited to take part in a 30-minute-long semi-structured interview to learn in more depth about their views on the intervention, their participation in study, their experience completing the 'CanRisk online training programme' (see section 5.2), their thoughts about the CanRisk output used to discuss the risk assessment results with women, and time/costs associated with the implementation of CanRisk (Appendix 26 – Participant information sheet for healthcare professionals and practice staff; Appendix 27 – Interview schedule for healthcare professionals). Separate consent will be taken prior to the interviews (Appendix 28 – Consent form for interviews with healthcare professionals and practice staff).

One practice manager and/or research lead per research site (if different from the GP taking part in the study) will also be invited to complete the NPT NoMAD survey completed by GPs and take part in a 20-minute-long semi-structured interview to gain more in-depth understanding about how the study was implemented in their practice and the time/costs associated with the implementation of CanRisk (Appendix 26 – Participant information sheet for healthcare professionals and practice staff; Appendix 29 – Interview schedule for practice staff; Appendix 28 – Consent form for interviews with healthcare professionals and practice staff). Practice managers and research leads who decline the invitation for their practices to join the study will also be asked to provide their main reason for declining. These data will be collected by the CRN on an online Google form adapted specifically for this study (Appendix 30 – Practice staff decliner form).

We will additionally aim to audio or video record a sub-set of 20 (approximately 15 minute-long) General Practice appointments with women at increased risk to reflect on how risk, management options and next steps are discussed in practice, as well as the time spent on CanRisk appointments. Additionally, for patients who agree to have their appointment recorded, we plan to administer a survey to patients immediately after their GP consultation, in order to explore their experience of the appointment. Starting at the beginning of the study and until we have recorded sufficient consultations, the breast cancer risk assessment results letters for women at increased risk will include information about our wish to audio or video record GP appointments (Appendix 13 – Moderate and high-risk results letter and Appendix 12 - Near population risk with additional risk factors results letter). This will only be sent to those women who consent to be contacted about having their consultation recorded on the main consent form (Appendix 5 – ICF Main). Women interested in this element of the study will be asked to read the Participant Information Sheet and complete the online consent form. For participants that do not respond to the invitation for consultation recording, a single reminder will be emailed to them after 3 days of them receiving their risk letter. Participants will be asked to contact the research team with the appointment details once they have made an appointment at their GP surgery. In addition, we will request this information from the GP too. For participants that consent to the recording but do not provide an appointment time within 3 days, a reminder will be sent to the patient to request the appointment time if available. For women who agree to have their consultation recorded, the research team will make arrangements with the GP practice to audio/video record the consultation depending on the type of appointment booked (in person or telephone) and if the patient has consented to audio and/or video recordings. At the beginning and end of the consultation, the GP will check that the patient still consents to the recording. If a patient expresses a desire to withdraw their consent, this will be recorded by the research team. The researcher will also confirm with the GP at the end of the consultation that they still consent to the recording. If a patient withdraws consent for the recordings to be used, the recordings will be destroyed (Appendix 31 – Participant information sheet for audio and video recording for patients; Appendix 32 – Participant information sheet for audio and video recording for healthcare professionals; Appendix 33 – Consent form for audio and video

recordings for patients; Appendix 34 – Consent form for audio and video recordings for healthcare professionals).

3.9 Withdrawal of consent and death

If participants wish to withdraw from the study no further data will be collected on them, although we will keep all data collected to that point. All patients will be free to withdraw consent during the qualitative interviews without giving a reason. They will be asked if they consent to us using the data collected prior to withdrawal. If they do not, then it will not be used and the data collected will be destroyed prior to analysis. We will also ask participants to let the research team know if they move to a different GP surgery before receiving their risk results. Participants moving to a practice that is not registered with the study before receiving their risk results will be withdrawn from the study and will not be sent their results. All patient withdrawals will be recorded in the study database by the research team. If a participant expresses a wish to withdraw from the study to the GP practice, the site team will inform the research team as soon as possible via email to the study NHS email account.

If a site becomes aware that a participant has died, this will be reported to the research team as soon as possible via email to the study NHS email account. The research team will record all reported deaths in the study database.

3.10 Safety Reporting

Related Serious Adverse Events occurring between the time the participants consent to the study and the time that their final result letter is sent to them will be recorded and reported in line with Good Clinical Practice (GCP).

A Serious Adverse Event (SAE) is defined as any untoward medical occurrence in a patient or trial participant that:
- results in death,
- is life-threatening*,
- requires hospitalisation or prolongation of existing hospitalisation**,
- results in persistent or significant disability or incapacity, or
- consists of a congenital anomaly or birth defect.

Other 'important medical events' may also be considered serious if they jeopardise the participant or require an intervention to prevent one of the above consequences

* A life- threatening event, this refers to an event in which the participant was at risk of death at the time of the event; it does not refer to an event which hypothetically might have caused death if it were more severe.
** Hospitalisation is defined as an in-patient admission, regardless of length of stay. Hospitalisation for pre-existing conditions, including elective procedures do not constitute an SAE.

Reporting
SAEs that are considered to be related to study participation will be reported to the research team from consent until 30 days after the date of the participant's final results letter. Site teams should aim to report related SAEs within 24 hours of becoming aware of the event. SAEs must be reported to the research team by completing the SAE form provided in the ISF to the study email address.

Grading
The grading of reported events will be assessed by a delegated investigator (as documented on the site delegation log) and recorded in the patient medical records.

The following grades will be used:

- Mild – The adverse event does not interfere with the participant's daily routine, and does not require further intervention; it causes slight discomfort
- Moderate – The adverse event interferes with some aspects of the participant's routine, or requires further intervention, but is not damaging to health; it causes moderate discomfort
- Severe – The adverse event results in alteration, discomfort or disability which is clearly damaging to health

Causality
The causality of reported events will be assessed by a delegated investigator (as documented on the site delegation log) and recorded in the patient medical records.

The following options will be used:

- Related – A causal relationship between the intervention and the event is at least a reasonable possibility, i.e., the relationship cannot be ruled out.
- Not related – There is no reasonable possibility of a causal relationship between the intervention and the event.

Onward reporting
Upon receipt of an SAE report, the research team will assess the event for expectedness. For this study, there are no expected events.

The report will be sent by the research team to the CI for causality assessment. If the assessment of the site investigator and the CI do not match, the worst case will be used (e.g. If the site assesses the event to be related and the CI assessed the event to be unrelated, the event will be assumed as related). The CI will also provide a statement of assessment of implication on safety.

If an event is confirmed to be both unexpected and related, the research team will report the event to the REC within 15 days of the report being received. The event will be reported using the HRA 'Report of Serious Adverse Event' form.

The research team will notify the Sponsor of any related unexpected SAEs requiring reporting to the REC.

All steps will be documented in the Trial Master File (TMF) by the research team.

3.11 Sample size

We aim to recruit 1,000 women into this study. Based on distributions of known risk factors in the UK, it is estimated that approximately 17% (170) of these women will be at above population-level risk[24]. This will enable us to estimate:

- the proportion of eligible women in each practice who consent to take part in the study with an accuracy of +/- 1.1% (width of the 95% CI) based on an estimate of 20%
- the proportion of eligible women in each practice who consent who complete the risk assessment with an accuracy of +/- 2.5% based on an estimate of 80%
- the proportion of women who consent who are at moderate or high risk on initial CanRisk assessment in general practice with an accuracy of +/- 2.5% based on an estimate of 17%
- the proportion of those at moderate or high risk who attend for an appointment with a GP and who are referred to secondary/tertiary care with an accuracy of +/- 5.7% based on an estimate of 80%

Based on estimated baseline means and standard deviations (SD) of 11.08 (SD 3.85) for STAI and 12.1 (SD 3.5) for the Lerman cancer worry scale-R[25], and allowing for a 40% loss to follow-up, 1,000 participants will also enable us to detect a change in anxiety or worry of 0.5 SD or greater at each time point with 95% confidence and 90% power in both women at population level risk and in the subgroup identified at moderate or high risk. Approximately 100 women aged between 40-49 are currently referred to the regional genetics clinic per year based on their family history from across Cambridgeshire and Peterborough through routine care and approximately 50 identified as at moderate or high risk. With approximately 65,000 women aged 40-49 across Cambridgeshire and Peterborough, this equates to approximately 1.5 per 1,000 registered women aged 40-49. Based on an estimated 17% of women being at above-population level risk, with a 20% response rate, we anticipate a referral rate of up to 34 per 1,000 registered women aged 40-49 years at participating practices if all eligible women are invited. One thousand participants will enable us to detect an estimated increase in the proportion of registered women identified as at moderate and high risk through proactive multifactorial risk assessment across all the participating practices compared with the 12 months prior to the study with a 95% confidence interval of +/- 0.005 (5 per 1,000 registered women). One thousand participants will also provide an adequate number of women, both population level risk and moderate/high risk, from whom to recruit for qualitative interviews and to complete the mixed-methods process evaluation.

To recruit 1,000 women, we estimate we will need to invite 5,000 women from between five to eight general practices. This is based on the following assumptions: the average size of the population registered with a General Practice is 12,000; approximately 6% of the population registered with GP practices across Cambridgeshire and Peterborough are aged between 40-49 years and eligible for inclusion in this study; in the only study in the UK to invite women to complete a breast cancer risk assessment (based on a family history questionnaire), uptake into the study was 16%[26]. We estimate uptake of 20% into this study.

The additional referrals anticipated both to the regional genetics clinic and for enhanced screening amongst those women estimated to be at moderate or high risk can be absorbed within the relevant services without any impact on existing referral pathways.

3.12 Analysis

3.12.1 Quantitative data analysis

Descriptive statistics will be used to summarise characteristics of the eligible population and study population at baseline, recruitment and retention rates, satisfaction and understanding of test results, healthcare utilisation, and intention to change behaviour overall and by risk group. Means and standard deviations will be used for normally distributed continuous variables, medians and interquartile range for non-normally distributed continuous variables, and numbers and percentages for categorical variables. Where reported, all proportions and differences in proportions will be presented with 95% confidence intervals. Statistical significance will be $p<0.05$. All analyses will be conducted in STATA.

Uptake of breast cancer risk assessment

In addition to reporting the proportion of eligible women who take up the offer of breast cancer risk assessment and the proportion of women eligible women who complete the risk assessment, we will use logistic regression to assess the participant level characteristics associated with completion of the risk assessment amongst those consenting to the study, reporting both unadjusted and adjusted odds ratios (OR) with 95% confidence intervals. Chi-squared tests will also be used to compare the proportions of those invited who take up the offer of breast cancer risk assessment between

demographic subgroups (i.e. age, ethnicity and IMD) using aggregate data from each participating practice. Statistical significance will be $p<0.05$.

The quantity of self-report data using MyCanRisk
The amount of information completed for non-family history risk factors (e.g. height, weight, alcohol, women's health) alongside the number of family members reported in the pedigree will be summarised using descriptive statistics. T-tests/ANOVA will be used to assess differences in the amount of information provided between demographic subgroups (i.e. age, ethnicity and IMD). Statistical significance will be $p<0.05$.

Psychological and behavioural impact of multifactorial breast cancer risk assessment
For continuous outcomes (anxiety, worry, risk perception, recall of risk information, and intentions) we will use analysis of covariance to calculate change at four weeks, three months and six months post scan within each risk group, adjusting for clustering between each GP practice. We have chosen this approach initially rather than fitting linear mixed effects models as we are primarily interested in differences between groups at each time point rather than differences in changes over time. We will, however, consider fitting linear mixed effects models with interactions between time and risk group. Any differences between the three risk groups on analysis of covariance will be tested using an F-test, followed by estimation of the comparison between those at near population level risk and those at moderate or high risk based on the CanRisk assessment. All models will be adjusted for age, ethnicity, education level, deprivation, baseline EQ-5D and general practice. The size of the change between groups will be interpreted by comparison with the standard deviation (SD), with the criteria for clinically relevant change being a change of 0.5 SD [27]. An F-test will also be used to test for interactions between the risk groups and age, family history of breast cancer, numeracy, health literacy, beliefs about breast cancer, decision making, time orientation, intolerance of uncertainty and responding "not anxious or depressed" versus "slightly/moderately/severely or extremely anxious or depressed" on EQ-5D at baseline. Analyses will be repeated within the subgroups where the p-value for interaction is <0.05.

3.12.2 Process evaluation and qualitative data analysis

Descriptive statistics will be used to analyse the results from the questionnaires. Content analysis will be used to analyse the short open text responses from non-participants (women and practice managers and or research leads). A fidelity checklist based on the training provided to GPs will be used alongside the video recordings from healthcare appointments to assess the adherence of GPs to the protocol during consultations with women at moderate or high risk. Audio from the video recorded appointments will be transcribed verbatim before analysis. Qualitative data from semi-structured interviews will be analysed using a mix of inductive and deductive Thematic Analysis. All interviews will be audio recorded and transcribed verbatim before analysis. The inductive analysis will look for emergent themes relevant to the study aims and the deductive thematic analysis will be organised around the seven dimensions of the Theoretical Framework of Acceptability[28]. Findings from interviews with participants, non-participants and clinicians will be compared, looking for concordant and discordant themes.

4. Data Management

4.1 Data storage and access

The handling, storage and transfer of all data collected and generated during the project will be managed according to recommended good practice, as described by the Digital Curation Centre (https://www.dcc.ac.uk/dmps) and in accordance with ICH GCP and current data protection legislation.

Identifiable participant data received electronically will be held in strictest confidence by the research team at University of Cambridge, with access limited to the research team by two-factor authentication. Users with access to the database will be logged by the research team on a Database User Log. Data will be stored on the University of Cambridge Secure Research Computing Platform (SRCP). SRCP is ISO 27001 certified and is NHS Security and Protection Toolkit compliant.

With the exception of communications between the research team, Yourgene (or other suitable provider), GP surgeries and within the research team about specific participants and their visits, care or data, any data transferred will not contain personal identifiable information.

The saliva samples will be stored prior to processing in laboratories within Strangeways Research Laboratory (SRL), Cambridge accessible only to members of the Cancer Genetic Epidemiology Group. Samples will be batched for sending to Yourgene (or other suitable provider) for processing. Samples will be posted by courier with complete tracking. After analysis, the residual DNA will be returned to the research team and stored in freezers within SRL, Cambridge. Residual DNA of participants who have not consented to future research, will be destroyed upon receipt from YourGene (or other suitable provider). All freezers have independent alarms that notify of any incidence of going out of temperature range, so that sample integrity is maintained. In the event of freezer failure, we have spare -80C freezer capacity to move the samples into. Temperature data is logged and held on the system. Professor Antonis Antoniou will be the custodian of the samples and sample management procedures and governance are managed by Mr Stephen Clarke as Person Designate for the Human Tissue license at the SRL.

Transfer of data between the researchers and Yourgene (or other suitable provider) will be performed via SFTP to a client specific location or as password protected documents. The personal information on study participants will be registered and stored on a sample receipt log that is password protected. Only those staff at Yourgene (or other suitable provider) requiring access to the data will be given the password. A unique participant ID will be used during sample processing and all run traceability worksheets and reports containing the ID will be stored in a password protected folder. The IDAT data generated from the GSA array analysis at Yourgene is stored on a stand-alone laptop (not networked to a company server) and has restricted, password protected access. The Next Generation Sequencing (NGS) data generated at Yourgene is analysed and stored within a secure Illumina BaseSpace Sequencing Hub account. Security specifics for BaseSpace can be found here: https://emea.illumina.com/content/dam/illumina-marketing/documents/products/whitepapers/basespace-sequence-hub-security-and-privacy-white-paper-970-2016-020.pdf Access to the Yourgene Genomic Services BaseSpace account and the data it contains is restricted by use of specific credentials with limited staff members having access.

Personal identifiable data, data within the Yourgene BaseSpace Sequencing Hub and the IDAT file location will be retained until the end of study, namely six months after the last participant has received their results letter, in accordance with the Yourgene Genomic Services sample and data retention policy. DNA samples will then be returned to the research team for storage as above and all study data destroyed. IDAT data will be transferred from YourGene to University of Cambridge via SFTP and be stored in a secure University of Cambridge electronic location to be used in this or future research. The IDAT data will not include any personal identifiable information. Similar secure storage, retention and destruction approaches will be employed with other suitable provider used for the genetic sample analysis.

Transfer of personal data within the research team and between the research team and GP surgeries will be done via NHS.net email (Appendix 36 – Email correspondence with participants).

Link-anonymised data will be transferred electronically between members of the research team, following local data security policies. Any transfer of link-anonymised or identifiable data between the research team and the practices will be done via NHS mail.

The recordings and anonymised transcriptions from interviews and consultations will initially be saved onto a University of Cambridge encrypted computer and then uploaded to the University of Cambridge SRCP. After being uploaded to the SRCP, the local copy will be deleted. For transcription. recordings will be securely uploaded (via SFTP or other encrypted method) to an external transcription company who will have signed a confidentiality agreement.

4.2 Data anonymisation

All potentially eligible participants will be allocated a unique participant ID number at the point of being invited to the study and will be identified by this number on all study related documentation throughout the course of the study and data analysis process. NHS number will also be used as a unique identifier. All study data will be link-anonymised at source wherever possible, or as soon as possible afterwards if applicable. The link file connecting the unique study ID number with personal data will be stored securely and separately to the trial data.

Interviewees will be given a pseudonym, so they remain anonymous in the reporting of the qualitative data. All personal data will be securely destroyed 12 months after the end of the study unless otherwise stated below.

The following personal identifiable data will be collected:

**Data**	**Purpose**	**Storage/Deletion Details**
Name	Sending of baseline questionnaire, MyCanRisk and follow up questionnaires by email. Sending of saliva sample kits by post.<br><br>Collection of data via NHS Digital services (HES and NCRAS) in this and future studies.	Entered by participants at consent and stored in the safehaven database.<br><br>Will be retained for 10 years after closure of the study for use in future research.
Address	Sending of saliva sample kits by post. Sending of baseline questionnaire, MyCanRisk and follow up questionnaires if post is selected as preferred method of contact as consent.	Entered by participants at consent and stored in the safehaven database.
Post code	Sending of saliva sample kits by post. Sending of baseline questionnaire, MyCanRisk and follow up questionnaires if post is selected as preferred method of contact as consent.<br><br>Calculation of Index of Multiple Deprivation (IMD).<br><br>Collection of data via NHS Digital services (HES and NCRAS) in this and future studies.	Entered by participants at consent and stored in safehaven database.<br><br>Will be retained for 10 years after closure of the study for use in future research.

Email address	Sending of baseline questionnaire, MyCanRisk and follow up questionnaires by email.	Entered by participants at consent and stored in the safehaven database.
Date of birth	Calculation of participant's risk via CanRisk tool. Generation of MyCanRisk URL.<br><br>Collection of data via NHS Digital services (HES and NCRAS) in this and future studies.	Entered by participants on consent form and stored in the safehaven database.<br><br>Will be retained for 10 years after closure of the study for use in future research.
NHS Number	Tracking and identification of participants.<br><br>Collection of data via NHS Digital services (HES and NCRAS) in this and future studies.	Provided by participating sites via NHS email following participant consent.<br><br>Will be retained for 10 years after closure of the study for use in future research.
Signature	Informed consent.	Entered by participants at consent and stored in the safehaven database and secure servers.

5. Study management

5.1 Participating Sites

The trial will take place in 5-8 GP practices in the Cambridgeshire and Peterborough areas. Sites will be sent a Study Summary by the CRN and be asked to complete an Expression of Interest Form. Sites that are interested will be supported by the CRN and will contact the research team to begin site set up. In addition, remote meetings may be conducted as required by either the practices or the research team. Where practices decline to participate in the study, reasons for declining will be collected where possible via the CRN practice decliner form.

Participating Sites will be required to provide the following documents as part of the site up process:

- Completed site staff delegation log and contact information
- Principal Investigator (PI) CV and GCP training certificate
- Localised Organisation Information Document (OID)

The research team will perform a remote or in-person site initiation visit (SIV) for all sites prior to activation. Site staff will also be required to complete the study training modules outlined in section 5.2. Sites will be provided with a paper ISF.

Once all activation items are complete, the research team will provide sites with a confirmation of site activation letter which will then permit sites to begin inviting patients to the study.

Sites are responsible for the maintenance of the ISF throughout the duration of the study, ensuring timely record keeping and filing of study documentation.

The research team will request final documents at the end of the trial for the purposes of site closure. Archiving of the trial at site cannot begin until site closure has been confirmed.

5.2 Site Training

In addition to the SIV, the research team will provide training to GPs within each participating practice to support consultations with women either with additional risk factors within their family history or at estimated moderate or high-risk. The main aim of the 'CanRisk online training programme' is to increase primary care professionals' knowledge and confidence to conduct multifactorial breast cancer risk prediction and it is composed of two tiers.

Tier one will be compulsory for GPs taking part in this study and will be composed of educational videos. Tier two will signpost trainees to optional existing relevant educational resources and a selection of scientific journal articles for further learning.

The content covered in the educational videos was developed following a scoping review of the training needs of healthcare professionals in primary care. Specific content plans for each video were shared with the members of our primary care expert advisory panel and refined using their feedback (Appendix 14 – CanRisk training educational videos content plan).

The videos (around 8 minutes each) will feature experts in cancer genetics, risk prediction, risk communication and primary care and will cover the following four learning objectives: 1) to develop background knowledge about the CanRisk tool; 2) to recognise how genetic testing and multifactorial risk prediction for breast cancer works in practice; 3) to identify the outcomes and management options for each risk group identified through multifactorial breast cancer risk prediction; and 4) to communicate the results of multifactorial breast cancer risk prediction in primary care.

To complement this, the research team has selected the following e-learning modules from the NHS England e-learning for healthcare website (https://www.e-lfh.org.uk/programmes/genomics-in-the-nhs/) for tier two of the training (optional): Genomics in Healthcare; Investigating the Genome Part 1: The Process; Public Health Masterclass in Genomics; Dominant, Recessive and Beyond: How Genetic Conditions are Inherited; Taking and Drawing a Genetic Family History and; Talking Genomics: tips and tools for communicating with patients.[1]

5.3 Sponsor

The Cambridge University Hospitals NHS Foundation Trust will act as sponsor for the trial jointly with the University of Cambridge.

5.4 Funding

Funder: Cancer Research UK (Ref. PPRPGM-Nov20\100002).

5.5 Portfolio Adoption

The study is adopted onto the UKCRN Research Portfolio.

5.6 Peer Review

This study has undergone external peer review by the research advisory committee, organised by the Sponsor and within the CanRisk programme team. The broader CanRisk programme, including this study, has been reviewed by the CR UK scientific committee as part of the funding application.

5.7 Public participation in the research

This study has been designed in collaboration with the CanRisk Patient and Public Involvement/Engagement (PPIE) group.. To date, our patient/public partners have been involved in securing the funding from CRUK, designing the study and reviewing the study documentation. Over the course of the study, we will meet with our patient/public partners on a regular basis, along with our primary care advisory group. We will also seek additional advice/guidance on issues that arise throughout the study as necessary. Once the data collection is complete, we anticipate that our patient/public partners will support data interpretation following initial analysis and will help to disseminate the findings once the study is complete.

5.8 Risk Assessment

A detailed assessment of risk will be made prior to study activation. Risk management decisions will be documented in the risk assessment, signed by the CI and filed in the TMF.

Prior to submission of any substantial amendment to the REC, the CI will provide a statement of risk assessment and any detail of changes to the original risk assessment.

5.9 Oversight Committees

The core research team will meet regularly throughout the study. Co-investigators will be updated regularly through various methods including the CanRisk programme meeting.

The Trial Management Group (TMG) will include the CI, Senior Researcher, Study Manager and Study Coordinator at a minimum. The TMG will be responsible for overseeing the study and will meet regularly, at least monthly. The TMG will review recruitment figures, SAEs and substantial amendments to the protocol prior to submission to the REC, as applicable.

.

All TMG activity will be documented in the TMF along with TMG membership details.

5.10 Protocol Deviations and Incidents

5.10.1 Protocol Deviations

Protocol deviations are minor unintended departures from the expected conduct of the study protocol and Standard Operating Procedures (SOP), which do not impact the participant’s safety or compromise the integrity of the study data (eg. A study visit being outside the window defined in the protocol). Protocol deviations will be recorded in the Protocol Deviation Log in the ISF.

5.10.2 Incidents

An incident encompasses any untoward event or significant deviation from the protocol. An Incident Report Form may be required for more serious protocol deviations. Completed forms will be sent to the research team. Upon receipt, the research team will log the details on the central Incident Log and file in the TMF.

The research team will also use the central Incident Log to document any incidents occurring within the research team, data management team and central laboratories.

5.10.3 Protocol Violation

A reportable protocol violation is a breach which is likely to effect to a significant degree a) the safety or physical or mental integrity of the participants of the trial or b) the scientific value of the trial.

If a site believes a protocol violation has occurred, they will notify the research team within 24hrs of becoming aware by email, complete a study Incident Report and add details to the site Protocol Deviation Log in the ISF.

5.11 Monitoring

The study Monitoring Plan outlines the timelines and methods of monitoring at site and centrally. The degree of monitoring will be proportionate to the risks associated with the study. Risk will be assessed on an ongoing basis as according to section 5.8.

5.12 Trial Master File

The TMF will be in an electronic format, hosted on secure University of Cambridge servers with restricted access according to the central delegation log.

Management of the TMF is outlined in the TMF Plan.

5.13 Central Delegation of Duties and Training

All research team staff performing study activity will be documented on the central delegation log and signed off by the CI. Updates to the log will be made according to changes in staffing or delegation of duties.

All site team staff performing study activity will be appropriately trained and have up to date GCP training. Records of training will be filed in the TMF.

5.14 Dissemination

Findings from the study will be reported in open-access papers in peer-reviewed journals and presented at national and international conferences. We will also provide a lay summary of the findings on the study website.

5.15 Data sharing

After the results have been analysed and published by the research team, anonymised quantitative data arising from the MyCanRisk app and study questionnaires will be stored in and made publicly available through the University of Cambridge repository (https://www.repository.cam.ac.uk/). Qualitative data (anonymised transcripts from the interviews and consultations) will be stored within the Primary Care Unit and will be available to researchers upon request through the University of Cambridge repository (https://www.repository.cam.ac.uk/). Researchers will be required to complete a Data Access Agreement that will indicate the criteria for data access and conditions for research use and will incorporate privacy and confidentiality standards to ensure data security.

5.16 Ethical review and reports

The Sponsor will ensure that the study protocol, PIS, ICF, GP letters and submitted supporting documents have been approved by the appropriate REC prior to any participant recruitment. The protocol, all other supporting documents including amendments, will be documented and submitted for ethical approval as required. Amendments will not be implemented prior to receipt of the required approvals.

Before any site can enrol participants into the study, the CI or designee will ensure that the appropriate approvals have been issued, and NHS Confirmations of Capacity and Capability (C&C) and Sponsor green lights are in place.

For any amendments to the study, the CI or designee, in agreement with the Sponsor, will submit information to the appropriate body in order for them to issue approval for the amendment. The CI or designee will work with sites to confirm ongoing C&C for the study.

It is the responsibility of the CI, or designee, to produce the annual progress reports (APR) when required; an APR will be submitted to the Sponsor and REC within 30 days of the anniversary date on which the favourable opinion was issued, and annually until the study is declared ended.

Within 90 days after the end of the study, the CI will ensure that the REC is notified that the study has finished. If the study is terminated prematurely, those reports will be made within 15 days after the end of the study.

Within one year after the end of the study, the CI will submit the Final Report with the results, including any publications/abstracts, to the Sponsor and to the REC and HRA.

All correspondence with the Sponsor, REC and HRA will be retained in the TMF.

5.17 Insurance and Indemnity

Cambridge University Hospitals NHS Foundation Trust, as a member of the NHS Clinical Negligence Scheme for Trusts, will accept full financial liability for harm caused to participants in the study through the negligence of its employees and honorary contract holders. There are no specific arrangements for compensation should a participant be harmed through participation in the study, where no-one has acted negligently.

The University of Cambridge will arrange insurance for negligent harm caused as a result of protocol design and for non-negligent harm arising through participation in the study.

5.18 Archiving

The Sponsor, CI and each participating site recognise that there is an obligation to archive study-related documents at the end of the study. The CI confirms that they will archive the TMF according to Sponsor requirements and in line with all relevant legal and statutory requirements.

The PI at each participating site agrees to archive their respective site's study documents in line with all relevant legal and statutory requirements.

Study documents will be archived for a minimum of 5 years from the study end. The research team will inform the site teams when archiving can be initiated.

7. References

1. National Institute for Health and Care Excellence. Familial breast cancer: classification, care and managing breast cancer and related risks in people with a family history of breast cancer. CG164. Published online 2017.
2. Gray E, Donten A, Karssemeijer N, et al. Evaluation of a Stratified National Breast Screening Program in the United Kingdom: An Early Model-Based Cost-Effectiveness Analysis. *Value in Health*. 2017;20(8):1100-1109. doi:10.1016/j.jval.2017.04.012

3. Evans DGR, Donnelly LS, Harkness EF, et al. Breast cancer risk feedback to women in the UK NHS breast screening population. *British Journal of Cancer*. 2016;114(9):1045-1052. doi:10.1038/bjc.2016.56
4. Evans DG, Brentnall AR, Harvie M, et al. Breast cancer risk in young women in the national breast screening programme: Implications for applying NICE guidelines for additional screening and chemoprevention. *Cancer Prevention Research*. 2014;7(10):993-1001. doi:10.1158/1940-6207.CAPR-14-0037
5. Usher-Smith JA, Hindmarch S, French DP, et al. Proactive breast cancer risk assessment in primary care: a review based on the principles of screening. *Br J Cancer*. 2023;(January). doi:10.1038/s41416-023-02145-w
6. French DP, Astley S, Astley S, et al. What are the benefits and harms of risk stratified screening as part of the NHS breast screening Programme? Study protocol for a multi-site non-randomised comparison of BC-predict versus usual screening (NCT04359420). *BMC Cancer*. 2020;20(1):1-14. doi:10.1186/s12885-020-07054-2
7. Smith SG, Sestak I, Forster A, et al. Factors affecting uptake and adherence to breast cancer chemoprevention: A systematic review and meta-analysis. *Annals of Oncology*. 2016;27(4):575-590. doi:10.1093/annonc/mdv590
8. Yang X, Eriksson M, Czene K, et al. Prospective validation of the BOADICEA multifactorial breast cancer risk prediction model in a large prospective study. *J Med Genet*. Published online 2022:1-10. doi:10.1136/jmg-2022-108806
9. Pal Choudhury P, Brook MN, Hurson AN, et al. Comparative validation of the BOADICEA and Tyrer-Cuzick breast cancer risk models incorporating classical risk factors and polygenic risk in a population-based prospective cohort of women of European ancestry. *Breast Cancer Research*. 2021;23(1):1-5. doi:10.1186/s13058-021-01399-7
10. Schwartz LM, Woloshin S, Black WC, Welch HG. The role of numeracy in understanding the benefit of screening mammography. *Annals of Internal Medicine*. 1997;127(11):966-972. doi:10.7326/0003-4819-127-11-199712010-00003
11. Watson M, Lloyd S, Davidson J, et al. The impact of genetic counselling on risk perception and mental health in women with a family history of breast cancer. *British Journal of Cancer*. 1999;79(5/6):868-874.
12. Marteau TM, Bekker H. The development of a six-item short-form of the state scale of the Spielberger State-Trait Anxiety Inventory (STAI). *The British journal of clinical psychology / the British Psychological Society*. 1992;31 (Pt 3):301-306.
13. Rabin R, de Charro F. EQ-5D: a measure of health status from the EuroQolGroup. *Annals of Medicine*. 2001;33(5):337-343.
14. Carleton RN, Norton MAPJ, Asmundson GJG. Fearing the unknown: A short version of the Intolerance of Uncertainty Scale. *Journal of Anxiety Disorders*. 2007;21(1):105-117. doi:10.1016/j.janxdis.2006.03.014
15. Crockett RA, Weinman J, Hankins M, Marteau T. Time orientation and health-related behaviour: Measurement in general population samples. *Psychology and Health*. 2009;24(3):333-350. doi:10.1080/08870440701813030
16. Rowlands G, Khazaezadeh N, Oteng-Ntim E, Seed P, Barr S, Weiss BD. Development and validation of a measure of health literacy in the UK: The newest vital sign. *BMC Public Health*. 2013;13(1):9-14. doi:10.1186/1471-2458-13-116
17. Simon AE, Forbes LJL, Boniface D, et al. An international measure of awareness and beliefs about cancer: development and testing of the ABC. *BMJ open*. 2012;2(6). doi:10.1136/bmjopen-2012-001758
18. Cella D, Hughes C, Peterman A, et al. A brief assessment of concerns associated with genetic testing for cancer: The Multidimensional Impact of Cancer Risk Assessment (MICRA) questionnaire. *Health Psychology*. 2002;21(6):564-572. doi:10.1037//0278-6133.21.6.564

19. Lerman C, Trock B, Rimer BK, Jepson C, Brody D, Boyce A. Psychological side effects of breast cancer screening. *Health Psychology*. 1991;10(4):259-267. doi:10.1037//0278-6133.10.4.259
20. Mills K, Paxton B, Walter FM, Griffin SJ, Sutton S, Usher-smith JA. Incorporating a brief intervention for personalised cancer risk assessment to promote behaviour change into primary care: a multi-methods pilot study. *BMC Public Health*. 2021;21(205). doi:10.1186/s12889-021-10210-3
21. Morris NS, MacLean CD, Chew LD, Littenberg B. The Single Item Literacy Screener: Evaluation of a brief instrument to identify limited reading ability. *BMC Fam Pract*. 2006;7. doi:10.1186/1471-2296-7-21
22. Sekhon M, Cartwright M, Francis JJ. Development of a theory-informed questionnaire to assess the acceptability of healthcare interventions. *BMC Health Services Research*. 2022;22(1):1-12. doi:10.1186/s12913-022-07577-3
23. May C, Finch T, Mair F, et al. Understanding the implementation of complex interventions in health care: the normalization process model. *BMC health services research*. 2007;7:148. doi:10.1186/1472-6963-7-148
24. Lee A, Mavaddat N, Wilcox AN, et al. BOADICEA: a comprehensive breast cancer risk prediction model incorporating genetic and nongenetic risk factors. *Genet Med*. 2019;21(8):1708-1718. doi:10.1038/S41436-018-0406-9
25. Foster C, Evans DGR, Eeles R, et al. Predictive testing for BRCA 1/2: Attributes, risk perception and management in a multi-centre clinical cohort. *British Journal of Cancer*. 2002;86(8):1209-1216. doi:10.1038/sj.bjc.6600253
26. Qureshi N, Dutton B, Weng S, et al. Improving primary care identification of familial breast cancer risk using proactive invitation and decision support. *Fam Cancer*. 2021;20(1):13-21. doi:10.1007/s10689-020-00188-z
27. Norman GR, Sloan JA, Wyrwich KW. Interpretation of changes in health-related quality of life: the remarkable universality of half a standard deviation. *Med Care*. 2003;41(5):582-592. doi:10.1097/01.MLR.0000062554.74615.4C
28. Sekhon M, Cartwright M, Francis JJ. Acceptability of healthcare interventions: An overview of reviews and development of a theoretical framework. *BMC Health Serv Res*. 2017;17(1):1-13. doi:10.1186/s12913-017-2031-8

8. Protocol Appendices

8.1 Appendix 1: Document Appendices

Appendix 1. Primer text message to women – no longer in use
Appendix 2. Invitation letter to women
Appendix 3. Participant information sheet
Appendix 4. Reminder letter
Appendix 5. Consent form
Appendix 6. Baseline questionnaire
Appendix 7. Screenshots from MyCanRisk
Appendix 8. Saliva sample kit covering letter
Appendix 9. Near population risk results letter
Appendix 10. Breast awareness leaflet
Appendix 11. Example of CanRisk output
Appendix 12. Near population risk with additional family history risk factors results letter
Appendix 13. Moderate and high risk results letter
Appendix 14. CanRisk training educational videos content plan
Appendix 15. Referral proforma for GPs
Appendix 16. One-month follow-up questionnaire
Appendix 17. Three-month follow-up questionnaire
Appendix 18. Six month follow-up questionnaire
Appendix 19. Participant information sheet for interviews with women
Appendix 20. Interview schedule for women
Appendix 21. Consent form for interviews for women
Appendix 21b – Consent form for FU interviews with women
Appendix 22. Decliner form
Appendix 23. Interview schedule for women who decline
Appendix 24. PGS decliner form
Appendix 25. Healthcare professional post-study questionnaire
Appendix 26. Participant information sheet for healthcare professionals and practice staff
Appendix 27. Interview schedule for healthcare professionals
Appendix 28. Consent form for interviews with healthcare professionals and practice staff
Appendix 29. Interview schedule for practice staff
Appendix 30. Practice staff decliner form
Appendix 31. Participant information sheet for audio and video recording for patients
Appendix 32. Participant information sheet for audio and video recording for healthcare professionals
Appendix 33. Consent form for audio and video recordings for patients
Appendix 34. Consent form for audio and video recordings for healthcare professionals
Appendix 35. Acceptance/decline slips – no longer in use
Appendix 36. Email correspondence with participants
Appendix 37. Data flow
Appendix 38. Near population risk results letter - updated info
Appendix 39. Near population risk with additional risk factors results letter - updated info
Appendix 40. Moderate and high risk results letter - Updated info
Appendix 41. Post-consultation survey
Appendix 42. Saliva re-sampling kit covering letter

8.2 Appendix 2: Summary of Study Activities

Activity	Participant	Site	Research Team
Invitation and Reminders		X	
Consent	X		
Data Transfer		X[a]	
Baseline Questionnaire	X		
MyCanRisk[b]	X		X
Saliva Samples	X[c]		X[c]
Risk Results			X
Clinical Appointment[d]		X	
Follow Up Questionnaires	X[e]		X[e]
Data Collection	X[f]		X[g]
Staff Qualitative Interviews			X
Staff Questionnaires			X
Participant Qualitative Interviews			X
SAE Reporting		X[h]	
Withdrawal/Death Reporting		X[h]	

[a] Sites will provide NHS number for all consented participants

[b] Links to access MyCanRisk will be sent by the research team to the participant. The participant will complete MyCanRisk remotely.

[c] The research team will send saliva sample kits to the participant. The participant will return the saliva sample to the research team for processing. Saliva sample results will then be sent to the site teams by the research team.

[d] Participants identified as population risk with additional risk factors and those at medium/high risk must be invited for a clinical appointment with the GP within 14 days of the risk results letter.

[f] Follow up questionnaires will be sent by the research team to the participant. The participant will complete the questionnaire remotely.

[g] Site teams will provide the research team anonymised data exports at the end of recruitment as requested by the research team. These will include site level aggregate data and outcome data for consented participants.

[h] SAE, withdrawal and death reporting is performed by emailing the research team.

8.3 Appendix 3: Glossary of Key Terms

APR	Annual Progress Reports
ANOVA	Analysis of Variance
AUC	Area Under the receiver operating characteristic Curve
BC	Breast Cancer
BOADICEA	Breast and Ovarian Analysis of Disease Incidence and Carrier Estimation Algorithm
BRCA	Breast Cancer gene
C&C	Confirmation of capacity and capability
CI	Chief Investigator
CRN	Clinical Research Network
CRUK	Cancer Research UK

E/O	Expected to Observed
FDR	First Degree Relative
FH	Family History
GCP	Good Clinical Practice
GSA	Global Screening Array
GP	General Practitioner
HES	Hospital Episode Statistics
HRA	Health Research Authority
HRT	Hormone Replacement Therapy
ICF	Informed Consent Form
ICO	Information Commissioner's Office
IDAT	Intensity Data File
IMD	Index of Multiple Deprivation
IRAS	Integrated Research Application System
ISF	Investigator Site File
MICRA	Multidimensional Impact of Cancer Risk Assessment
NCRAS	National Cancer Registration and Analysis Service
NGS	Next Generation Sequencing
NICE	The National Institute of Health and Care Excellence
NIHR	National Institute for Health and Care Research
NoMAD	Normalization Process Theory Measure
NPT	Normalisation Process Theory
OID	Organisation Information Document
OR	Odds Ratio
PI	Principal Investigator
PIS	Patient Information Sheet
PPIE	Patient and Public Involvement/Engagement
PGS	Polygenic Risk Score
PV	Pathogenic Variant
REC	Research Ethics Committee
SAE	Serious Adverse Event
SD	Standard Deviation
SFTP	Secure File Transfer Protocol
SIV	Site Initiation Visit
SOP	Standard Operating Procedure
SRL	Strangeways Research Laboratory
STAI	State-Trait Anxiety Inventory
TFA	Theoretical Framework of Acceptability
TMF	Trial Master File
TMG	Trial Management Group
TSC	Trial Steering Committee
UKCRN	UK Clinical Research Network

8.4 Appendix 4: Summary of Changes

Version number	Date	Summary
3.0	25/10/2023	N/A – first approved version
3.1	18/03/2024	- Update to database details

4.0	20/06/2024	- Update to study contacts<br>- Update to study processes<br>- Addition of safety reporting guidance<br>- Removal of primer messages<br>- Update to age range and data collection timeframe in outcomes<br>- Addition of withdrawal and death reporting instructions<br>- Addition of personal identifiable data handling information<br>- Addition of study management sections<br>- Update to study timetable<br>- Addition of contents page<br>- Addition of abbreviation section<br>- Addition of summary of changes table<br>- Formatting
4.1	13/09/24	- Addition of optional survey on GP consultation experience<br>- Formatting and typos<br>- Added a few missing appendices<br>- Amended Figure 2
4.2	03/02/25	- Amended PPIE representatives<br>- Added upper initial search limit of 48<br>- Added several reminders<br>- Updated further exclusion criteria reflecting a previously updated PIS and ICF (no change to actual exclusion criteria)<br>- Updated timelines<br>- Removed study timetable<br>- Removed references to TSC<br>- Update to study processes<br>- Update to data management<br>- Updated study figure
4.3	10/04/25	- Reformatted section on interviews, clarifying the amount of participants to be recruited, as well as the sub-sets to be recruited.<br>- Clarified consent process for post-study questionnaires for GPs
4.4	08/08/25	- Inclusion of follow-up interview process<br>- Inclusion of other providers for PGS and related processing<br>- Additional text relating to withdrawal in cases of moving practice during the study
4.5	30/10/25	- Removed requirement of clinical accreditation for labs processing saliva samples.
